# TLC–Densitometry for Determination of Omeprazole in Simple and Combined Pharmaceutical Preparations

**DOI:** 10.3390/ph15081016

**Published:** 2022-08-18

**Authors:** Wioletta Parys, Alina Pyka-Pająk

**Affiliations:** Department of Analytical Chemistry, Faculty of Pharmaceutical Sciences in Sosnowiec, Medical University of Silesia in Katowice, 41-200 Sosnowiec, Poland

**Keywords:** TLC, densitometry, omeprazole, diclofenac sodium, pharmaceutical preparation

## Abstract

TLC combined with densitometry was used and chromatographic conditions developed to separate omeprazole and diclofenac sodium from their potential impurities. The development of the TLC–densitometry method is based on the elaboration of new chromatographic conditions allowing for the simultaneous determination of omeprazole and diclofenac sodium in a pharmaceutical preparation. Identification and quantification of omeprazole in simple and combined (with diclofenac) pharmaceutical preparations was performed on silica gel 60F_254_ using one mobile phase: chloroform–methanol–ammonia (36:4:0.60, *v*/*v*). Diclofenac sodium was determined in the presence of omeprazole after 2D separation on silica gel using two mobile phases of the first phase of chloroform–methanol–ammonia (36:4:0.60, *v*/*v*) and the second mobile phase cyclohexane–chloroform–methanol–glacial acetic acid (6:3:0.5:0.5 *v*/*v*). The developed method is simple, economical, specific, precise, accurate, sensitive, and robust, with a good range of linearity for the quantification of omeprazole and diclofenac sodium. TLC in combination with densitometry can be used as an effective analytical tool for quality control and quantitative determination of omeprazole in simple and combined pharmaceutical preparations containing diclofenac sodium. TLC in combination with densitometry can be recommended for the analysis of omeprazole and diclofenac sodium in the absence of HPLC or spectrophotometer in the laboratory or to confirm results obtained with other analytical techniques.

## 1. Introduction

**Figure 1:**
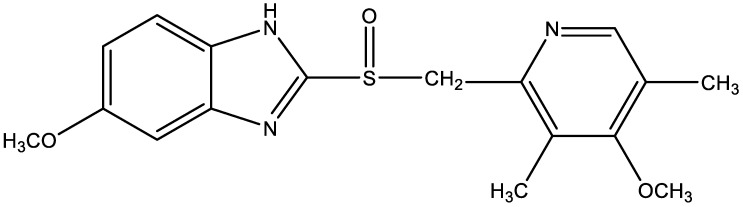
Structural formula of omeprazole.

Proton-pump inhibitors (PPIs) found clinical application over 35 years ago and have been considered indispensable ever since, as they show particular safety and efficacy in the treatment of a wide range of disorders related to gastric hyperacidity. Although all drugs of this class act in a similar way (they inhibit the active secretion of hydrochloric acid in the parietal cells), there are slight differences between PPIs with regard to their pharmacokinetic properties and clinical indications. Nevertheless, each of them is effective in the treatment of gastroesophageal reflux disease and uncomplicated or complicated peptic ulcer disease [1]. These drugs (PPIs) can be found in simple and combined preparations along with nonsteroidal anti-inflammatory drugs, such as ketoprofen and diclofenac. By lowering the pH of the gastric contents, they reduce any damage, erosions, and ulcers of the gastric mucosa associated with taking nonsteroidal anti-inflammatory drugs (NSAIDs) [2]. Chemically, omeprazole is 5-methoxy-2-[(RS)-[(4-methoxy-3,5-dimethylpyridin-2-yl)methyl]sulfinyl]-1H-benzimidazole [3] (Figure 1).

Omeprazole crosses cell membranes and exhibits weak base properties (it is acid-labile). After leaving the stomach, like the other proton-pump inhibitors, it is absorbed in the proximal part of the small intestine. The circulatory system transports the drug to the parietal cells of the stomach, which actively produce gastric acid, and it concentrates in the secretory tubules [1,4,5,6,7].

**Figure 2:**
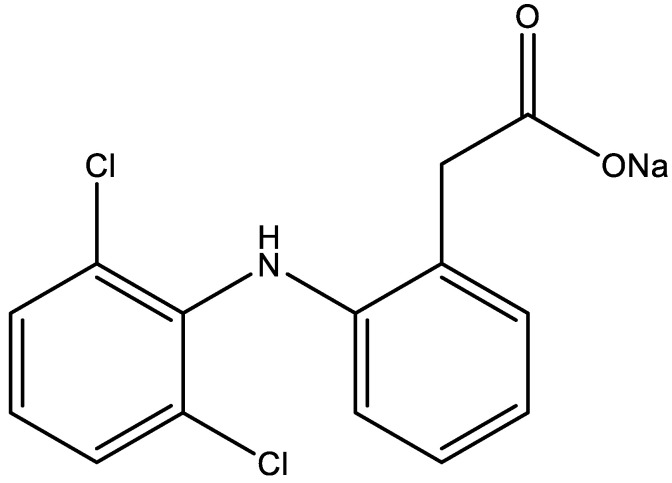
Structural formula of diclofenac sodium.

Diclofenac belongs to the group of NSAIDs, and more specifically is a derivative of phenylacetic acid. It was registered in 1979. Chemically, it is o-N-(2,6-dichlorophenyl)aminophenylacetic acid, and its summary formula is C_14_H_10_Cl_2_NO_2_ [3] (Figure 2).

This drug is a powerful anti-inflammatory, antipyretic, and pain reliever. It is absorbed at lightning speed and completely from the digestive system. It quickly reaches its maximum concentration in the blood serum and remains there for up to several hours. The indications for the use of the drug are: all kinds of inflammation of the musculoskeletal system, neuralgia, severe pain of various origins, gout attacks, contusions, joint damage, and local inflammatory changes [7,8]. The selected physicochemical properties of omeprazole and diclofenac sodium [9,10,11,12,13] are presented in Table 1.

Chromatographic techniques are most often used in drug analysis, as evidenced by pharmacopoeial descriptions. Omeprazole is most often analyzed by TLC/HPTLC [14,15,16,17,18,19,20,21,22,23,24,25], HPLC [23,26,27,28,29,30,31,32,33,34,35,36,37,38,39,40], spectrophotometry [41,42,43], LC-MS/MS [44,45], GC [46], voltammetry [46], and polarography [47]. Few studies have investigated the possibility of determining omeprazole in the presence of its degradation products [18,25,29,31,39]. Agdaba et al. [18] developed a method for the determination of omeprazole and pantoprazole along with their impurities with omeprazole sulfone and N-methyl pantoprazole by means of HPTLC on silica gel plates. The mobile phase was a mixture of chloroform–propanol–25% ammonia–acetonitrile (10.8:1.2:0.3:4, *v*/*v*/*v*/*v*). It allowed the separation of the abovementioned four tested compounds. The limit of detection for omeprazole was 0.005 mg/mL [18]. Jha et al. [25] determined omeprazole in pharmaceutical preparations by HPTLC on plates precoated with silica gel 60F_254_ and using a mobile phase of chloroform–methanol (9:1, *v*/*v*). Omeprazole was subjected to stressful conditions (acid-, base-, oxidation-, wet heat-induced degradations, as well as photochemical degradation). The applied chromatographic conditions allowed for the separation of omeprazole from its degradation products in acidic and alkaline environments, hydrogen peroxide, and omeprazole irradiated with solar and UV radiation and heated dry [25]. Flor et al. [29] determined omeprazole along with its sulfone derivative in pharmaceutical preparations using HPLC-UV. The LOD of omeprazole was 0.4 mg/mL. The HPLC-UV method was found to be suitable for quality-control and stability studies of omeprazole in pharmaceutical preparations [29]. In another experiment, HPLC was used to ensure appropriate determination of degradation products and impurities of ketoprofen and omeprazole in a complex oral solid dosage form [31]. Chromatographic separation was achieved on a Phenomenex Luna C18 column (150 × 4.6 mm, 5 µm) with a gradient elution using a mixture of potassium dihydrogen phosphate buffer and acetonitrile at a flow rate of 0.8 mL/min. The study was monitored at a wavelength of 233 nm for ketoprofen impurities and at 305 nm for the omeprazole impurities using a UV detector. To prove the stability of the method, the drug product was subjected to hydrolytic, oxidative, photolytic, humidity, and thermal conditions. The developed method was validated according to the current ICH guidelines and proved to be sufficiently precise, sensitive, and selective [31]. El-Sherif et al. [39] used RP-HPLC to determine lansoprazoles, omeprazoles, and pantoprazoles in the presence of their degradation products in an acidic environment. The analyses were performed on a C18 column using 0.05 M potassium dihydrogen phosphate–methanol–acetonitrile (5:3:2, *v*/*v*/*v*) as mobile phase. El-Sherif et al. [39] found that omeprazole is degraded into five products in an acidic environment.

Omeprazole was determined next to ketoprofen [19,22,31,32], aspirin [20,21,24,33], other drugs belonging to the PPI group [36,43], ondansetron [17,27], domperidone [30], aspirin, and salicylic acid [33]. Bhatt et al. [15] tested the TLC method to identify proton-pump inhibitors and accompanying drugs, including diclofenac sodium. Planar chromatographic separation was obtained using silica gel 60 F_254_ and a mobile phase consisting of toluene, isopropanol, acetone, and ammonia (5:2.3:2.5:0.2, *v*/*v*/*v*/*v*). The densitometric measurement was performed at λ = 290 nm. The method showed good linearity, as indicated by high values of correlation coefficients (≥0.9993). The limit of detection (for omeprazole) was set at 12.7 ng per spot, and the limit of quantification was 38.1 ng per spot. The developed method was sensitive, precise, and accurate [15]. Omeprazole and diclofenac sodium side by side were also determined spectrophotometrically [41,42] and by RP-HPLC [34]. However, none of these studies tested the presence of potential degradation products of omeprazole and diclofenac sodium or subject samples containing omeprazole and diclofenac sodium to stress conditions. Pharmacopoeia recommends liquid chromatography to determine the content of omeprazole in simple pharmaceutical preparations and diclofenac sodium also in simple pharmaceutical preparations. However, no pharmacopoeia describes the simultaneous determination of omeprazole and diclofenac sodium next to each other. As such, there is no pharmacopoeial method for determining omeprazole and diclofenac sodium next to each other.

This work is a continuation of the research concerning the use of TLC in combination with densitometry for drug analysis. Among other things, Parys et al. [48] investigated the stability and quantified diclofenac sodium in enteric tablets. The aim of this study was to use the TLC technique combined with densitometry for the determination of omeprazole in simple (omeprazole genoptim SPH and Bioprazol Bio Max), as well as of omeprazole and diclofenac sodium in combined (DicloDuo Combi) pharmaceutical preparations. The proposed methods were validated according to the accepted standards described in the ICH guideline [49], as well as Ferenczi-Fodor et al. [50].

The biggest novelty of the presented work is the possibility of determining omeprazole and diclofenac sodium next to each other in a sample using TLC combined with densitometry. The scientific literature so far has not described the method of TLC combined with densitometry for the simultaneous determination of omeprazole and diclofenac sodium.

## 2. Results and Discussion

### 2.1. Validation of TLC–Densitometry Method

The applied method has been fully validated and the validation results are presented in Figure 3, Figure 4, Figure 5, Figure 6, Figure 7 and Figures S1–S15 and in Table 2, Table 3, Table 4, Table 5 and Table 6, and in the following subsections.

#### 2.1.1. Selectivity of TLC–Densitometry Method

A total of ten mobile phases were tested in the search for chromatographic conditions enabling the separation of omeprazole from its degradation products and from diclofenac sodium and the degradation products of diclofenac sodium. As a result of the performed research, it was found that it is not possible to separate omeprazole, its degradation products, sodium diclofenac, or its degradation products using only one mobile phase. Therefore, in the TLC analysis, finally two mobile phases were used:

- chloroform–methanol–ammonia (36:4:0.60, *v*/*v*/*v*) (mobile phase IX).
- cyclohexane–chloroform–methanol–glacial acetic acid (6:3:0.5:0.5, *v*/*v*/*v*/*v*) (mobile phase X).

Mobile phase X in its composition contains glacial acetic acid. For this reason, the polarity of mobile phase X is greater than that of mobile phase IX.

**Figure 3:**
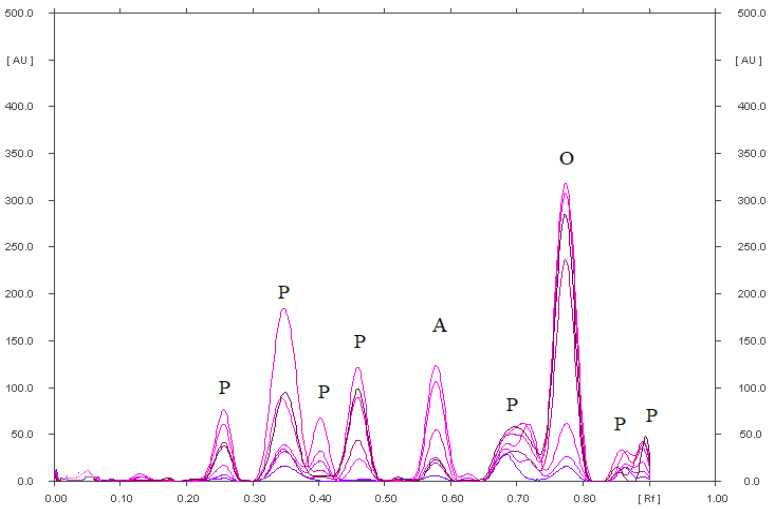
Densitogram of omeprazole (4 µg) in an acidic solution, which after heating was separated on silica gel using a mobile phase chloroform–methanol–ammonia (36:4:0.60, *v*/*v*/*v*); where: O-omeprazole, A-omeprazole related compound A and P-unidentified omeprazole degradation products.

It was found that mobile phase IX with the composition chloroform–methanol–25% ammonia (36:4:0.60, *v*/*v*/*v*) causes diclofenac sodium and its degradation products to remain almost at the start. They form one spot with a value of R_F_ = 0.05 (Figures S1–S4). A densitogram of a standard of diclofenac is shown in Figure S5. This mobile phase made it possible to perfectly separate the abovementioned substances from omeprazole and its degradation products. In addition, its use allowed for the separation of omeprazole from its degradation products, as confirmed in Figure 3 and Figures S6–S9. Table 2 contains the R_F_ values of omeprazole and its degradation products formed under various stress conditions. The most (eight) degradation products were found in the acidic environment (Figure 3, Table 2). Seven degradation products formed in an omeprazole solution in an alkaline environment (Figure S6) and in a methanolic omeprazole solution with the addition of hydrogen peroxide (Figure S7), which was heated for 90 min at a temperature of 80 °C. Under these three stress conditions, an omeprazole degradation product with the R_F_ value of 0.60 (±0.02) was identified: omeprazole-related compound A (omeprazole sulfone, 5-methoxy-2-{[(4-methoxy-3,5-dimethyl-2-pyridinyl)methyl] sulfonyl}-1H-benzimidazole) (Table 2 and Figure 3 and Figures S6 and S7). The fewest degradation products of omeprazole were formed in a methanolic solution of omeprazole with the addition of physiological saline heated for 90 min at a temperature of 80 °C (Figure S8) and in a methanolic solution of omeprazole irradiated with UV radiation at λ = 254 nm for 90 min (Figure S9). Two degradation products of omeprazole were found in both solutions. The results concerning the stability of omeprazole obtained in this study partially confirm the studies of other scientists [25,31,39,51]. All [25,31,39,51] showed unanimously that omeprazole shows high instability at low pH. Omeprazole is degraded to the most degradation products in an acidic environment (eight) based on the analysis of the densitograms by Jha et al. [25], which is consistent with this research. However, five degradation products of omeprazole were obtained in the presence of hydrogen peroxide and under the influence of sunlight [25]. For comparison, Koppala et al. [31] obtained the most omeprazole degradation products in an alkaline environment and in the presence of hydrogen peroxide (four degradation products) and in an acidic environment and under the influence of water hydrolysis (three degradation products). DellaGreca et al. [51] found that omeprazole is sufficiently stable at pH = 7.0 and above. However, a significant degradation of omeprazole takes place in a mildly acidic environment or under the influence of sunlight, which makes it impossible to determine it under these conditions [51]. Comparing the results of this work with those obtained by Koppala et al. [31], it can be assumed that the degradation products of omeprazole may be:

- in an acidic environment: 5-methoxy-2-{[(4-methoxy-3,5-dimethylpyridin-2-yl) methyl]sulfonyl}-1*H*-benzimidazole (omeprazole-related compound A), 5-methoxy-2-{[(4-methoxy-3,5-dimethyl-2-pyridinyl)methyl]sulfonyl}-1*H*-benzimidazole, 4-dihydro-1-(5-methoxy-1*H*-benzo[d]imidazol-2-yl) -3,5-dimethyl-4oxopyridine-2-carboxylic acid;
- in an alkaline environment: 5-methoxy-2-{[(4-methoxy-3,5-dimethylpyridin-2-yl) methyl]sulfonyl}-1*H*-benzimidazole (omeprazole-related compound A), 5-methoxy-2-{[(4-methoxy-dimethylpyridin-2-yl)methyl]sulfonyl}-1*H*-benzimidazole, 1,4-dihydro-1-(5-methoxy-1*H*-benzo[d]imidazol-2-yl)-3,5-dimethyl-4oxopyridine-2-carboxylic acid; and 5-methoxy-1*H*-benzimidozole-2-thiol;
- in solution with the addition of hydrogen peroxide (oxidation): 5-methoxy-2-{[(4-methoxy-3,5-dimethylpyridin-2-yl)methyl]sulfonyl}-1*H*-benzimidazole (omeprazole-related compound), 4-dihydro-1-(5-methoxy-1*H*-benzo[d]imidazole-2-yl)-3,5-dimethyl-4-oxopyridine-2-carboxylic acid.

The remaining degradation products should be considered unidentified.

Therefore, omeprazole can be separated from its degradation products as well as diclofenac sodium along with its degradation products using a mobile phase consisting of chloroform, methanol, and ammonia in a volume ratio of 36:4:0.6. It was thus concluded that the purity and quantification of omeprazole in the simple and combined drug in the presence of diclofenac and their degradation products can be tested using this mobile phase. However, this mobile phase should not be recommended for the determination of diclofenac, because this drug together with the degradation products formed one spot on the chromatogram (R_F_ = 0.05). When testing a drug containing omeprazole and diclofenac sodium, it should first separate diclofenac from omeprazole using the IX mobile phase. Omeprazole should be quantified under these conditions. A two-dimensional (2-D) development of chromatographic plate should be applied to quantify diclofenac, because diclofenac sodium (and its potential impurities) remains at the start of the chromatogram. Namely, the development must first be carried out in the mobile phase IX. Then dry the chromatographic plate, rotate it through an angle of 90° and then develop it with the mobile phase X, i.e., cyclohexane–chloroform–methanol–glacial acetic acid (6:3:0.5:0.5, *v*/*v*/*v*/*v*). The mobile phase X was previously used by Parys et al. [48] for the quantification of diclofenac sodium in enteric-coated tablets. It was shown that the mobile phase X is highly selective, because it enables the separation of diclofenac sodium from its degradation products (Table 3). Five degradation products of diclofenac sodium in an acidic environment in both the solutions heated for 5 h and 1.5 h. Five degradation products of diclofenac sodium were obtained when the solution was irradiated with UV radiation at λ = 254 nm (during 5 h) before application to the plate. However, six decomposition products of diclofenac sodium were obtained after applying the solution to the plate and then irradiating this solution on the plate [48]. The degradation product with R_F_ = 0.80 (±0.02) was identified as diclofenac-related compound A (1-(2,6-dichlorophenyl)-1,3-dihydro-2H-indol-2-one) [48], as confirmed by earlier studies by Krzek et al. [52], and Elzayat et al. [53]. Based on the available literature, it can be assumed that the unidentified degradation products of diclofenac sodium may be: 2,6-dichloro-*N*-(2-methylphenyl)aniline [54], 8-chloro-9*H*-carbazole-1-acetic aldehyde [55], 2-(9*H*-carbozl-1-yl)-2-hyroxyacetic acid, 2-oxo-2-(2-(phenylamino)phenyl)acetic acid, 1-(2,6-dichlorophenyl)-3-3-dihydroxyindolin-2-one, 2-(9*H*-carbazol-1-yl)acetic acid, 2-(8-chloro-9*H*-carbazop-1-yl)acetic acid [56], 1-(2,6-dichloro-*N*-(o-tolyl)anilin [57], N-phenyl-2,6-dichloroaniline, N-chloroacetyl-N-phenyl-2,6-chloroaniline [58].

**Figure 4:**
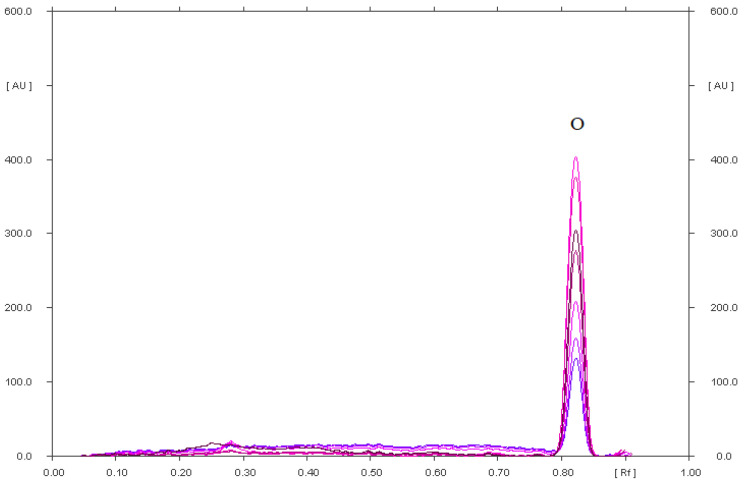
Densitogram of omeprazole (0.90 µg) from the extract of Biprazol Bio Max, which was analyzed on silica gel using a mobile phase: chloroform–methanol–ammonia (36:4:0.60, *v*/*v*/*v*).

**Figure 5:**
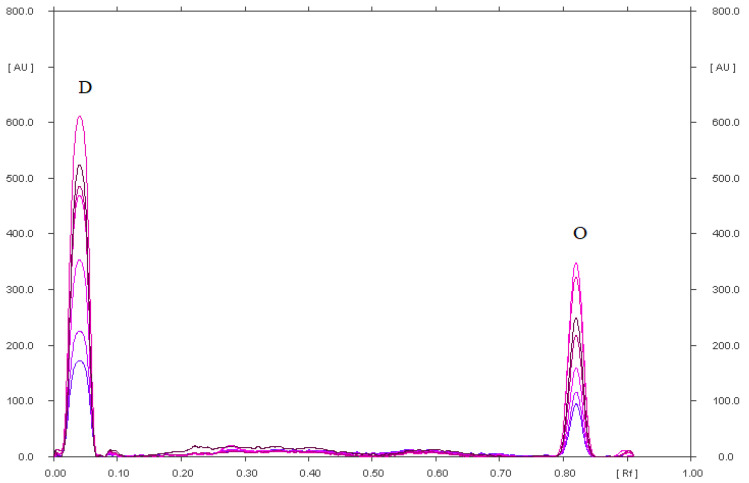
Densitogram of DicloDuo Combi extract, which was separated on silica gel using a mobile phase: chloroform–methanol–ammonia (36:4:0.60, *v*/*v*/*v*); where: D-diclofenac sodium (3.38 µg), O-omeprazole (0.90 µg).

**Figure 6:**
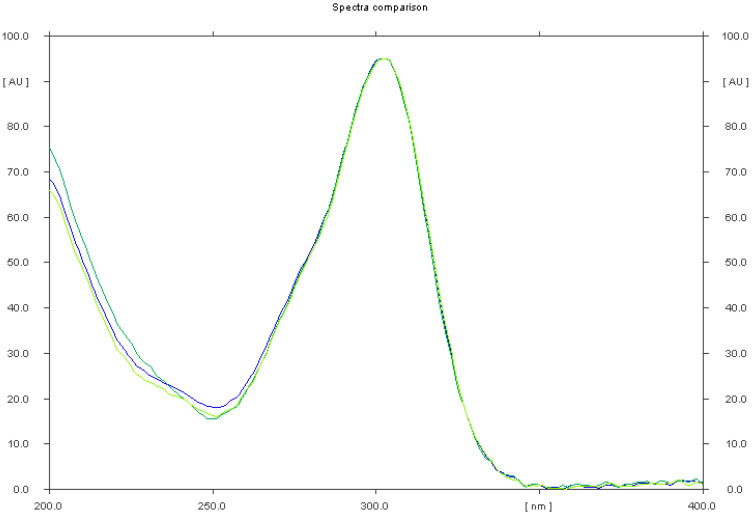
Comparison of the spectrodensitograms of omeprazole standard and omeprazole extracted from simple preparations (Omeprazole Genoptim SPH and Biprazol Bio Max).

Omeprazole was quantified in simple pharmaceutical preparations (omeprazole genoptim SPH and Biprazol Bio Max) and combined (DicloDuo Combi) using the IX mobile phase composed of chloroform–methanol–25% ammonia (9:1:0.15, *v*/*v*/*v*). TLC was found to be highly selective (Figure 4, Figure 5 and Figure S10) for the determination of omeprazole. The mean R_F_ value of the omeprazole standard (Figure S11) was 0.81 ± 0.03 and was consistent with the R_F_ value of omeprazole analyzed in the pharmaceutical formulations. The compatibility of the spectrodensitograms of the omeprazole standard with the spectrodensitograms of the omeprazole from capsules (Figure 6 and Figure S12) confirms the high selectivity of the developed TLC method.

**Figure 7:**
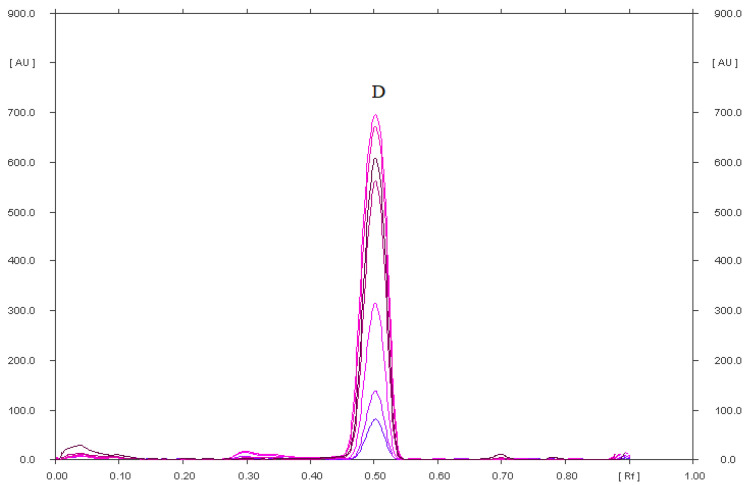
Densitogram of diclofenac sodium (14 µg) after two-dimensional (2-D) separation by TLC using two mobile phases, the first: chloroform–methanol–ammonia (36:4:0.60, *v*/*v*/*v*) and the second (after drying the chromatogram): cyclohexane–chloroform–methanol–glacial acid acetic acid (6:3:0.5:0.5, *v*/*v*/*v*/*v*).

**Figure 8:**
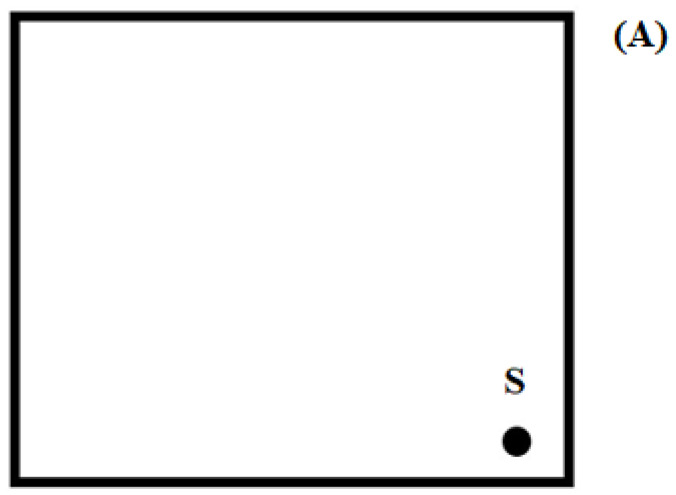

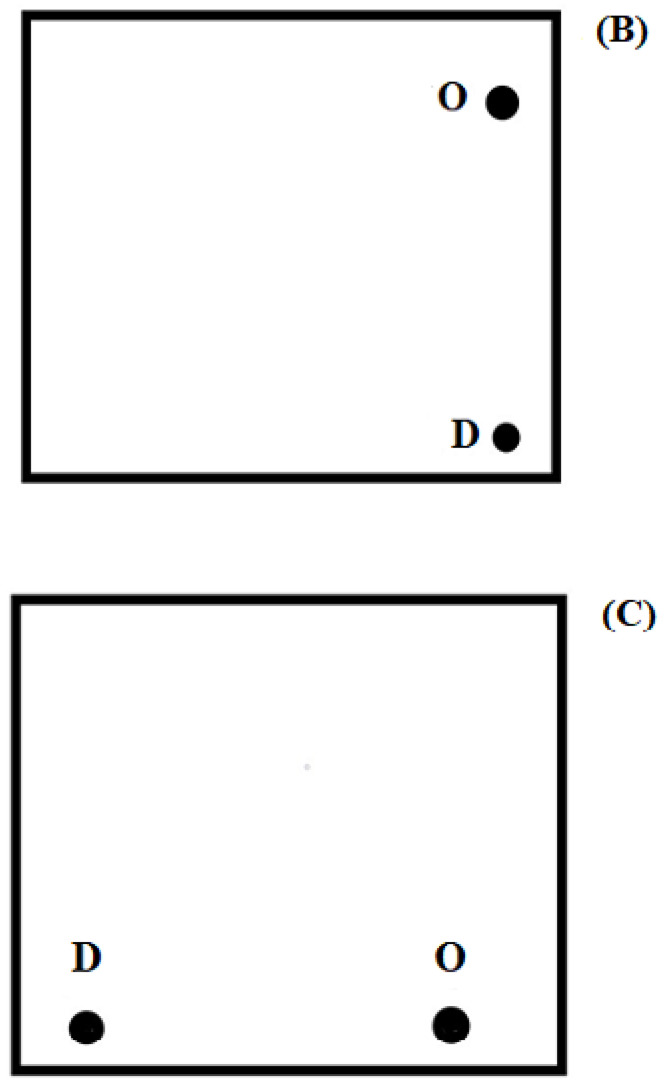
Chromatographic plate: (**A**) with DicloDuo Combi extract (S) or a mixture of diclofenac sodium and omeprazole standards (S) applied; (**B**) plate (**A**) after development in the mobile phase chloroform–methanol–ammonia (36:4:0.60, *v*/*v*/*v*); D—a spot representing diclofenac sodium, O—omeprazole; (**C**) the plate (**B**) was rotated by an angle of 90^o^ to develop in the mobile phase cyclohexane–chloroform–methanol–glacial acetic acid (6:3:0.5:0.5, *v*/*v*/*v*/*v*).

A pharmaceutical preparation called DicloDuo Combi was analyzed in this work. It consists of drugs from separate groups, i.e., diclofenac sodium and omeprazole. Diclofenac has anti-inflammatory and antipyretic properties. It is a valuable medicinal substance used in the treatment of moderate and severe inflammation accompanied by pain. However, it has a detrimental effect on the gastric mucosa, which is why the manufacturer combined it with omeprazole in the studied preparation. It works by inhibiting the secretion of hydrochloric acid in the stomach, and therefore constitutes a protective barrier against the irritating effect of diclofenac. When analyzing the DicloDuo Combi preparation, the analysis of two-dimensional (2-D) development of the chromatographic plate was carried out after applying the extract of this drug on it. This development method is illustrated by the schemes shown in Figure 8. After applying the DicloDuo Combi extract on the plate, it was developed using the first mobile phase, i.e., chloroform–methanol–ammonia, in a volume ratio of 36:4:0.60. The densitogram (Figure 5) according to which omeprazole has the R_F_ = 0.81 was obtained by densitometric scanning of this plate. Therefore, no impurities that were degradation products of omeprazole were found in the studied preparation. Diclofenac sodium and any impurities form a single spot with the R_F_ = 0.05 (Figures S1–S5) under these chromatographic conditions. The chromatographic plate (developed in the first mobile phase) was turned by 90° after drying and developed with the second mobile phase, i.e., cyclohexane–chloroform–methanol–glacial acetic acid (6:3:0.5:0.5, *v*/*v*/*v*/*v*) to check the purity of the drug and quantify diclofenac sodium. A densitogram showing a diclofenac sodium having the R_F_ = 0.47 ± 0.03 (Figure 7) was obtained by densitometric scanning of the second chromatogram. Therefore, no impurities, i.e., degradation products of diclofenac sodium, were found in the pharmaceutical preparation DicloDuo Combi. The mean R_F_ value of the diclofenac standard was consistent with the R_F_ value of diclofenac analyzed in the combined pharmaceutical formulation. The compatibility of the spectrodensitograms of the diclofenac standard with the spectrodensitograms of the diclofenac from capsules (Figure S13) confirms the high selectivity of the developed TLC method.

It was observed that excipients present in the formulation did not interfere with the omeprazole (Figure 4 and Figure S10) as well as with the omeprazole and diclofenac sodium peaks (Figure 5 and Figure 6). The peak purities of omeprazole from Omeprazole Genoptim SPH (Synoptis Pharma, Warsaw, Poland) and Bioprazol Bio Max (Biofarm, Wrocław, Poland), as well as omeprazole and diclofenac sodium from DicloDuo Combi (PharmaSwiss, Prague, Czech Republic) were also assessed by comparing the spectra obtained from omeprazole and diclofenac standards at the peak start (S), peak apex (M), and peak end (E) of spot (Figure 6 and Figures S12 and S13). It was found that r(S,M) > 0.999, and r(M,E) > 0.999 for all of the analyses performed by the TLC–densitometry technique. It should be stated that TLC combined with densitometry is highly selective for the determination of omeprazole and diclofenac sodium in capsules.

The results of the validation of the TLC–densitometry method are shown in Table 4, Table 5 and Table 6 and discussed in the following subsections.

#### 2.1.2. Linearity

The linear range was defined between the area of the spots [AU] and the concentration of omeprazole standard solutions and diclofenac sodium [µg/spot]. Standard concentrations are in the linear range from 0.04 to 1.00 µg/spot for omeprazole (Table 4) and from 5.0 to 15.0 µg/spot for diclofenac sodium (Table 5).

Calibration curves are presented in Figures S14A and S15A. These results confirm linearity of obtained calibration plots. On the basis of the calibration curves, the relative percentage error in determining the area of the chromatographic band (spot) was calculated for omeprazole and diclofenac sodium, respectively (Table S1). The calculated values of the relative percentage errors of the determination of the chromatographic band area (spot) were less than 3% for omeprazole and diclofenac sodium, respectively. The graphs of residuals against the concentration of omeprazole (Figure S14B) and diclofenac sodium (Figure S15B) were also plotted. It can be observed that the residuals were distributed above and below the zero residuals line, thus it confirms the linearity of proposed TLC methods for determination of omeprazole and diclofenac sodium in capsules

#### 2.1.3. Precision

The precision of the method was determined on the basis of the determined coefficient of variation CV [%] based on data from the measurement of chromatographic bands of omeprazole and diclofenac sodium. The coefficients of variation for intraday and interday precision ranged from 0.89% to 2.56% and 1.08% to 2.81% (Table 4) for omeprazole, respectively. The coefficients of variation for intraday and interday precision ranged from 1.13% to 2.42% and 2.38% to 2.78% (Table 5) for diclofenac sodium, respectively. The interday precision is characterized by higher % RSD values than the intraday precision. It is understandable that the value of interday precision exceeds the value of intraday precision, because its value is influenced by a much larger number of variables (e.g., measurement time, number of people performing the analysis). Intraday precision was performed by one analyst, and interday precision by two analysts.

In all cases, the value of the coefficient of variation did not exceed 3%, which allows us to conclude that the proposed analytical methods are precise in the determination of both omeprazole and diclofenac sodium in capsules.

#### 2.1.4. Accuracy

Recovery measurement was chosen to determine the accuracy of the proposed method, as no pharmacopoeia describes the method of simultaneous determination of omeprazole and diclofenac sodium. Also, pharmacopoeias do not specify what is the recommended certified reference material in the study of omeprazole and diclofenac sodium. The accuracy of the TLC methods combined with the densitometric analysis was assessed by measuring the drug recovery from capsules by adding 50%, 100% and 150% of the omeprazole standard and diclofenac sodium standard, respectively, to the drug samples, respectively.

The recovery of omeprazole ranged from 98.4% to 102.1% (Table 4). The recovery of diclofenac sodium ranged from 98.4% to 103.1% (Table 5). The CV [%] for the assays was less than 3% for both omeprazole and diclofenac sodium. This allows to conclude that the proposed analytical methods are accurate in the quantitative determination of omeprazole and diclofenac sodium in capsules.

#### 2.1.5. Limit of Detection (LOD) and Limit of Quantification (LOQ)

Two methods were evaluated. The first method for the determination of omeprazole in simple and combined pharmaceutical preparations after separation on silica gel 60F_254_ using the mobile phase chloroform–methanol–ammonia in a volume ratio of 36:4:0.60 was characterized by the limits of detection and quantification equal 0.009 µg/spot and 0.028 µg/spot for omeprazole. For the second method for the determination of diclofenac sodium in the presence of omeprazole after 2D separation on silica gel using two mobile phases: chloroform–methanol–ammonia in a volume ratio of 36:4:0.60 and the second mobile phase cyclohexane–chloroform–methanol–glacial acetic acid (6:3:0.5:0.5, *v*/*v*). These were characterized by the limits of detection and quantification equal 0.61 µg/spot and 1.84 µg/spot for diclofenac sodium. Particularly low LOD and LOQ values were obtained for omeprazole. However, both proposed methods are characterized by low LOD and LOQ for the determination of omeprazole and diclofenac sodium, which confirm sensitivity of the proposed methods.

#### 2.1.6. Robustness

The robustness of the developed method was investigated by analyzing the effect of small, deliberate variations in chromatographic conditions on the peak area of the examined drug sample. Table 6 shows the results of robustness of method for the five changed chromatographic parameters. The coefficients of variation of area of the chromatographic bands of omeprazole and diclofenac sodium, determined at the change of each of the chromatographic parameters, were less than 2% regardless of the tested pharmaceutical preparation. This indicates that the proposed methods are robust for the determination of omeprazole and diclofenac sodium.

#### 2.1.7. Quantitative Determination of Omeprazole in Simple and Combined Capsules

The content of omeprazole and diclofenac sodium in the studied pharmaceutical preparations was calculated using the calibration equations presented in Table 4 and Table 5, respectively. Statistic data concerning the results of assays are summarized in Table 7. The content of omeprazole in the range from 95.5% to 103.5% in relation to the content declared by the manufacturer was determined after the analysis of omeprazole by NP-TLC with densitometry after separation using chloroform–methanol–ammonia (36:4:0.60 *v*/*v*/*v*) as mobile phase. The content of diclofenac sodium was determined to be 98.7% in relation to the content declared by the manufacturer after analysis of diclofenac sodium by NP-TLC with densitometry after two-dimensional (2-D) separation using chloroform–methanol–ammonia (36:4:0.60, *v*/*v*/*v*) as first mobile phase and using cyclohexane–chloroform–methanol–glacial acetic acid (6:3:0.5:0.5, *v*/*v*/*v*/*v*) as second mobile phase. The *US Pharmacopoeia* allows the content of omeprazole and diclofenac sodium in capsules from 90.0% to 110.0% [59]. Thus, the determined contents of omeprazole and diclofenac sodium are within the range given in the pharmacopoeial monograph.

#### 2.1.8. Comparison of the Limit of Detection of Omeprazole and Diclofenac Sodium Obtained in This Work with Literature Methods

The limit of detection of analyzed omeprazole and diclofenac sodium by TLC technique in this work was compared with the available literature data, in which omeprazole and diclofenac sodium was also determined using various analytical methods. This comparison is summarized in Table 8. This table shows the limits of detection of omeprazole and diclofenac sodium in units in accordance with the cited publications. The limit of detection for omeprazole of 0.009 µg/spot (9 ng/spot; 0.18 µg/mL) was achieved in this study. The limit of detection obtained is comparable to the results obtained by other researchers or even better despite the use of more advanced HPTLC plates by other scientists [17,19,27,30] or the use of HPLC technique [24,29]. However, the TLC technique is most often less sensitive for the determination of omeprazole than HPLC and spectrophotometry, as can be seen from this comparison. In this study, worse LOD values for diclofenac sodium were obtained than others. This may be because two-dimensional (2-D) development should be used to determine diclofenac sodium in the presence of omeprazole. The use of two mobile phases in the 2-D analysis of diclofenac sodium contributes to a different blurring of chromatographic spots in relation to the performed analyzes using one mobile phase, and the change in the area of the chromatographic band affects the LOD value. This hypothesis is confirmed by the fact that in an earlier publication, Parys et al. [48] determined diclofenac sodium in enteric tablets using TLC but with one-dimensional development, and then obtained LOD equal to 0.28 µg/spot for diclofenac sodium. Nevertheless, the LOD value obtained is acceptable.

## 3. Material and Methods

### 3.1. Pharmaceutical Reference Standards and Chemicals

Omeprazole, omeprazole related compound A, (Pharmaceutical Secondary Standards; Certified Reference Materials, Sigma-Aldrich, St. Louis, MO, USA), and diclofenac sodium (European Pharmacopoeia (EP) Reference Standard, Sigma-Aldrich, USA) were used as standards. All chemicals and reagents for TLC method were analytical grade and were purchased from POCh (Gliwice, Poland).

### 3.2. Pharmaceutical Preparations

Simple pharmaceutical preparations containing only omeprazole were investigated, namely: omeprazole genoptim SPH (Synoptis Pharma, Warsaw, Poland) and Bioprazol Bio Max (Biofarm, Wrocław, Poland) in the form of intestinal capsules containing 20 mg of omepraazole. Moreover, the combined pharmaceutical preparation DicloDuo Combi (PharmaSwiss, Prague, Czech Republic) in the form of modified-release capsules was investigated; each capsule contained 75 mg of diclofenac sodium and 20 mg of omeprazole.

### 3.3. Preparation of Samples

After the capsule was opened, the contents of the capsule were weighed and then the amount containing 0.2 mg, 1.0 mg, and 1.8 mg of omeprazole as well as 12 mg, 20 mg and 28 mg of diclofenac sodium was weighed. Each weight was poured into a container containing four metal balls. It was then pulverized using the Ika Ultra Turrax Tube Drive for 9 min at a frequency of 9000 rpm, then 10 mL of methanol solution was added to perform the extraction. The parameters of the above-mentioned device were set at the frequency of 6000 rpm, and the extraction lasted 25 min. The obtained solutions were filtered to volumetric flasks (10 mL) and then made up to the mark with methanol. In this way, solutions of individual drugs were obtained, with concentrations in terms of omeprazole of 0.10 mg/5 mL, 0.50 mg/5 mL, 0.90 mg/5 mL and in terms of diclofenac sodium of 6.00 mg/5 mL, 10.00 mg/5 mL and 14.00 mg/5 mL.

### 3.4. Preparation of Standard Solutions

Standard solutions of omeprazole, omeprazole-related compound A, and diclofenac sodium were prepared in methanol.

### 3.5. Thin-Layer Chromatography

Chromatographic plates precoated with silica gel 60F_254_ with dimensions of 20 cm × 20 cm (E. Merck, Germany, # 1.05554), were cut to size 10 cm × 20 cm and used as the stationary phase for the study. Additionally, TLC silica gel 60F_254_ plates with dimensions of 10 cm × 20 cm (E. Merck, Germany, # 1.05570) were used for robustness tests. The plates were activated at 120 °C for 30 min. The solutions (5 µL) were spotted manually on the chromatographic plates using microcapillaries (Camag). Two mobile phases were used to carry out the experiment. The first one was a combination of chloroform, methanol, and ammonia in a volume ratio of 36:4:0.60 and was used for qualitative and quantitative studies of omeprazole (one-dimensional development was used). This was the case with simple pharmaceutical preparations containing omeprazole.

In the case of the analysis of the DicloDuo Combi preparation containing omeprazole and diclofenac sodium or a sample containing the standards of these substances and their possible degradation products, the procedure shown in Figure 8 was followed. After spotting the sample (Figure 8A) on the plate, it was developed using the first mobile phase: chloroform–methanol–ammonia (36:4:0.60, *v*/*v*/*v*). It was used to separate diclofenac sodium and its degradation products (they form one spot above the start) from omeprazole and its degradation products. The chromatogram shown schematically in Figure 8B was then obtained. After rotating the plate by 90° (Figure 8C), it is developed using the second mobile phase: cyclohexane–chloroform–methanol–glacial acetic acid (6:3:0.5:0.5, *v*/*v*/*v*/*v*). The second phase was used to separate diclofenac impurities from diclofenac sodium. The front of the mobile phase was always approximately 7.5 cm from the starting line after development of the plates. The abovementioned plates were dried at room temperature for 24 h (in a fume cupboard) before starting the densitometric analysis.

### 3.6. Densitometric and Spectrodensitometric Study

Densitometric and spectrodensitometric analysis were performed using a TLC Scanner 3 (Camag, Switzerland) operated in the absorbance mode and controlled by winCATS 1.4.2 software. The radiation source was a deuterium lamp emitting a continuous UV spectrum between 190 and 450 nm. Densitometric scanning was then performed at multiple wavelengths in the range of 220 to 400 nm, at wavelength intervals of 30 nm at each step. Finally, densitometric scanning was then performed at absorption maximum equal to 278 nm and 303 nm for diclofenac sodium and omeprazole, respectively. The slit dimensions were 12.00 × 0.40 mm, Macro. The optimal optical system was light; the scanning speed was 20 mm/s; the data resolution was 100 μm/step; the measurement type was remission; and the measurement mode was absorption.

The chromatographic bands obtained on the densitograms were investigated by spectrodensitometric analysis under the following conditions: the slit dimensions were 12.00 × 0.40 mm, Macro; the optimal system was resolution; the scanning speed was 20 nm/s; the data resolution was 1 nm/step; the initial wavelength was 200 nm, and final wavelength was 400 nm; the measurement type was remission; and the measurement mode was absorption.

### 3.7. Validation of the Thin-Layer Chromatography Method

The proposed NP-TLC–densitometry method was validated by specificity, linearity, accuracy, precision, limit of detection, limit of quantification, and robustness according to the ICH guidelines [49], according to the guidelines described by Ferenczi-Fodor et al. [50], and the *US Pharmacopoeia* [59].

#### 3.7.1. Specificity

The selectivity of the TLC method combined with densitometric analysis consisted in developing a chromatographic separation of omeprazole and diclofenac sodium from their degradation products. The stability of omeprazole and diclofenac sodium to acid and alkaline hydrolysis, oxidation, and photodegradation was tested. Diclofenac sodium solutions subjected to degradation were prepared according to the methodology provided earlier [48].

Methanolic standard solution of omeprazole was prepared by weighing and dissolving 40 mg of omeprazole in 10 mL of methanol. 1 mL of the standard solution was taken and 1 mL of 2 M HCl and 3 mL of methanol were added to it for acid hydrolysis, then 1 mL of the standard solution was taken and 1 mL of 2 M NaOH and 3 mL of methanol were added to it for alkaline hydrolysis. In the next stage of the experiment, the oxidation was carried out: 1 mL of the standard solution was taken and 1 mL of a 3% hydrogen peroxide solution and 3 mL of methanol were added to it for this purpose. The next solution was made by combining 1 mL of the standard solution and 1 mL of saline and 3 mL of methanol. These solutions were heated on a hotplate (Merck) at 80 °C for 90 min. The next solution was prepared by mixing 1 mL of the standard solution and 4 mL of methanol, which was irradiated with UV radiation (λ = 254 nm) for 90 min. The last solution (prepared in a similar way to the previous one) was used as a standard solution of omeprazole in the TLC studies.

In order to establish the optimal chromatographic conditions, the following mobile phases were investigated:

- chloroform–methanol 9:1 (*v*/*v*)
- chloroform–methanol 9:0.7 (*v*/*v*)
- chloroform–methanol 9:1.2 (*v*/*v*)
- chloroform–methanol 9:1.5 (*v*/*v*)
- chloroform–methanol–acetone 8.5:1:0.5 (*v*/*v*/*v*)
- chloroform–2-propanol–25% ammonia–acetonitrile 10.8:1.2:0.3:4 (*v*/*v*/*v*/*v*)
- chloroform–methanol–25% ammonia–acetonitrile 9:1:0.15:1 (*v*/*v*/*v*/*v*)
- chloroform–methanol–25% ammonia 9:0.7:0.10 (*v*/*v*/*v*)
- chloroform–methanol–25% ammonia 9:1:0.15 (*v*/*v*/*v*)
- cyclohexane–chloroform–methanol–glacial acetic acid 6:3:0.5:0.5 (*v*/*v*/*v*/*v*)

#### 3.7.2. Linearity and Range

The linearity of the TLC method was evaluated by analysis of standard solutions of omeprazole at concentrations 0.04, 0.08, 0.12, 0.16, 0.20, 0.30, 0.40, 0.50, 0.60, 0.70, 0.80, 0.90, and 1.00 mg/5 mL and diclofenac sodium at concentrations 5.00, 6.00, 7.00, 9.00, 10.00, 11.00, 12.00, 13.00, 14.00, and 15.00 mg/5 mL. The solutions (5 μL) were applied on the plate. The plates were developed using the abovementioned mobile phases (described in thin-layer chromatography section) and scanned. The experiments were performed in six different analyses.

#### 3.7.3. Accuracy

This parameter was evaluated by measurement of recovery, as no pharmacopoeia describes the method of simultaneous determination of omeprazole and diclofenac sodium. A proper amount of omeprazole and diclofenac sodium standards in the low, medium, and high regions of the calibration plots were added to powdered capsules of known active substance content. To the weights of simple drugs (omeprazole genoptim SPH and Bioprazol Bio Max) and the combined drug (DicloDuo Combi), which contained 8 mg of omeprazole, the following was added: (a) 4 mg; (b) 8 mg, (c) 12 mg of omeprazole standard. To the weights of combined drug (DicloDuo Combi), which contained 12 mg of diclofenac sodium, the following was added: (a) 6 mg, (b) 12 mg; (c) 18 mg of diclofenac sodium standard. The extraction was performed according to the procedure described in Section 3.3. The obtained diclofenac sodium solutions had the following concentrations: 9 mg/5 mL; 12 mg/5 mL, and 15 mg/5 mL. However, after dilution, the omeprazole solutions had the following concentrations: 0.6 mg/5 mL, 0.8 mg/5 mL, and 1.0 mg/5 mL. Next the samples were extracted and analyzed under the optimized conditions. The experiments were performed in six different analyses.

#### 3.7.4. Precision

Intraday and interday precision of the method was verified by analysis of three replicates of three sample solutions (methanol extracts of omeprazole and diclofenac sodium) at different concentrations under the same chromatographic conditions. The precision of the method was evaluated as the relative standard deviation (coefficient of variation, CV [%]).

#### 3.7.5. Limit of Detection (LOD) and Limit of Quantification (LOQ) Based on the Calibration Curves

Specific calibration curves were studied using samples containing omeprazole or diclofenac sodium in the range of the limit of detection, namely 0.04, 0.08, and 0.12 μg/spot for omeprazole and 2.00, 3.00 and 4.00 μg/spot for diclofenac sodium. The method of calculating the LOD and LOQ was described by Konieczka et al. [61].

#### 3.7.6. Robustness Study

The robustness of the proposed TLC–densitometry method was checked by evaluating the effect of small but deliberate changes of applied chromatographic conditions on the results, i.e., on the measured peak area of studied omeprazole and diclofenac sodium, respectively. Robustness was estimated by changing different chromatographic conditions in proposed procedure such as [48]:

- The kind of chromatographic plates (1.05554 and 1.05570): these aluminum plates were precoated with silica gel 60F_254_. The plates 1.05570 had a dimension 10 cm × 20 cm. The plates 1.05554 had a dimension 20 cm × 20 cm and before were cut to size 10 cm × 20 cm
- Mobile phase volume (50 mL ± 5%): 50 mL of mobile phase was used as standard. However, in the study of the robustness of the method, a mobile phase with a volume of 50 mL ± 5% was used, i.e., 47.5 mL and 52.5 mL
- Temperature of the activation of the plates at 120 (±5) °C
- Development distance (±5 mm)
- Time of saturation of chromatographic chamber (±5 min)

#### 3.7.7. Statistical Analysis

Statistical evaluation of the obtained results was performed with the use of the computer software Statistica 13.0.

## 4. Conclusions

The proposed TLC methods in combination with densitometry proved to be simple, economical, specific, precise, accurate, sensitive, and robust, with good ranges of linearity for the quantitative determination of omeprazole and diclofenac in pharmaceutical preparations. The development of the TLC–densitometry method used is based on the elaboration of new chromatographic conditions allowing for the simultaneous determination of omeprazole and diclofenac sodium in a pharmaceutical preparation. The developed conditions allow for the identification and quantification of omeprazole in simple and combined pharmaceutical preparations. Omeprazole was quantified on silica gel 60F_254_ after separation using chloroform–methanol–ammonia (36:4:0.60, *v*/*v*/*v*) as mobile phase. Omeprazole separates from its degradation products and from diclofenac sodium along with the degradation products of diclofenac sodium under these chromatographic conditions. The first step is to separate diclofenac from omeprazole using the abovementioned mobile phase when testing a drug containing omeprazole and diclofenac sodium. Omeprazole is quantified in these conditions, because diclofenac sodium and its potential impurities remain on the starting line. A two-dimensional (2-D) development was applied for the quantification of diclofenac. Namely, the first development was performed using the first mobile phase: chloroform–methanol–ammonia (36:4:0.60, *v*/*v*/*v*). The chromatographic plate was then dried, rotated by 90° and developed with the second mobile phase: cyclohexane–chloroform–methanol–glacial acetic acid (6:3:0.5:0.5, *v*/*v*/*v*/*v*). The content of omeprazole ranged from 95.5% to 103.5% and the content of diclofenac sodium was equal to 98.7% in pharmaceutical preparations in relation to the content declared by the manufacturer. The *US Pharmacopoeia* allows the content of omeprazole and diclofenac sodium from 90.0% to 110.0% in capsules [59]. Thus, the determined contents of omeprazole and diclofenac sodium are within the range given in the pharmacopoeial monograph. TLC in combination with densitometry can be used as an effective analytical tool for quality control and quantitative determination of omeprazole in simple and combined pharmaceutical preparations containing diclofenac sodium. TLC in combination with densitometry can be recommended for the analysis of omeprazole and diclofenac sodium in the absence of HPLC or a spectrophotometer in the laboratory, or to confirm the obtained results of the analysis with other analytical techniques. It could be suggested that the developed TLC–densitometry method may be used for the routine analysis of omeprazole in simple and combined pharmaceutical formulations. This method is suitable for analyzing of omeprazole as well as omeprazole combined with diclofenac sodium in pharmaceutical preparations without any interferences from additives present in pharmaceutical product. The biggest novelty of the presented work is the possibility of determining omeprazole and diclofenac sodium present next to each other in a sample. The scientific literature so far has not described TLC combined with densitometry for the simultaneous determination of omeprazole and diclofenac sodium.

## Figures and Tables

**Table 1:** Selected physicochemical properties of omeprazole and diclofenac sodium [9,10,11,12,13].

Physicochemical Property	Omeprazole	Diclofenac Sodium
Empirical formula	C_17_H_19_N_3_O_3_S	C_14_H_10_Cl_2_NNaO_2_
Molecular mass	345.4 g/mol	318.13 g/mol
Melting point	155 °C	283–285 °C
Solubility in water	0.359 mg/mL	21.3 g/L in 25 °C
pK_a_	9.29	4.15
logP_exp_	2.33	-
AlogP_s_	1.56	4.75
AClogP	2.48	4.62
xlogP2	0.60	3.85
xlogP3	2.23	4.71

**Table 2:** R_F_ values for omeprazole and omeprazole degradation products, R_F_ values for diclofenac and diclofenac degradation products ^(a)^ after analysis on silica gel using the chloroform–methanol–ammonia (36:4:0.60, *v*/*v*/*v*).

Stress Conditions	R_F_ of Omeprazole Degradation Products (P) and R_F_ of Omeprazole (O)
Omeprazole in an acidic environment heated at 80 °C for 90 min.	0.25; 0.35; 0.40; 0.45; 0.58 ^(b)^; 0.70; 0.85; 0.90 for P
	0.79 for O
Omeprazole with the addition of saline, heated at 80 °C for 90 min.	0.78; 0.89 for P
	0.82 for O
Omeprazole in an alkaline environment, heated at 80 °C for 90 min.	0.25; 0.34; 0.42; 0.57; 0.62 ^(b)^; 0.68; 0.72 for P
	0.79 for O
Omeprazole with the addition of hydrogen peroxide, heated at 80 °C for 90 min.	0.17; 0.40; 0.46; 0.51; 0.61 ^(b)^; 0.70; 0.79; 0.88 for P
	0.79 for O
Omeprazole in a methanolic solution irradiated with UV at λ = 254 nm for 90 min.	0.49; 0.70 for P
	0.81 for O
Reference solution	0.81 for O

^(a)^ R_F_ values for diclofenac sodium and its degradation products are equal 0.05. ^(b)^ omeprazole degradation product with the R_F_ = 0.60 (±0.02) was identified as Omeprazole Related Compound A (omeprazole sulfone).

**Table 3:** R_F_ values for diclofenac sodium and diclofenac degradation products^a)^ after analysis on silica gel using the cyclohexane–chloroform–methanol–glacial acid acetic acid (6:3:0.5:0.5, *v*/*v*/*v*/*v*) [48].

Stress Conditions	R_F_ of Degradation Products of Diclofenac Sodium (P) and R_F_ of Diclofenac Sodium (D)
Diclofenac sodium in an acidic environment heated at 90 °C for 90 min (1.5 h)	0.11, 0.38 0.63, 0.73, 0.80 ^(a)^ for P
	0.47 for D
Diclofenac sodium in an acidic environment, heated at 90 °C for 5 h	0.07, 0.11, 0.16, 0.73, 0.82 ^(a)^ for P
	0.49 for D
Diclofenac sodium in a methanolic solution irradiated with UV at λ = 254 nm for 5 h.	0.18, 0.37, 0.42, 0.65, 0.91 for P
	0.48 for D
Diclofenac sodium, which was exposed to UV radiation (λ = 254 nm) on silica gel for 5 h.	0.03, 0.21, 0.33, 0.41, 0.75, 0.91 for P
	or O

^(a)^ degradation product of diclofenac sodium with the R_F_ = 0.80 ± 0.02 was identified as diclofenac related compound A [1-(2,6-dichlorophenyl)-1,3-dihydro-2H-indol-2-one].

**Table 4:** Method validation data for the quantitative determination of omeprazole by NP-TLC with densitometry after separation using chloroform–methanol–ammonia (36:4:0.60 *v*/*v*/*v*).

Method Characteristic		Results		
Specificity		Specific		
Range [μg/spot]		0.04–1.00		
Linearity		A = 1907.3 (±47.4) + 13,491.4 (±86.7) · x *n* = 13; r = 0.999; s = 98.8; F = 24222; *p* < 0.0001		
Limit of detection (LOD) [μg/spot] Limit of quantification (LOQ) [μg/spot]		0.009 0.028		
For tablets Accuracy and precision				
		DicloDuo Combi	Omeprazole Genoptim SPH	Bioprazol Bio Max
Accuracy, *n* = 6				
for 50% standard added		R = 101.3%; CV = 2.21%	R = 99.6%; CV = 1.36%	R = 102.1%; CV = 2.01%
for 100% standard added		R = 98.4%; CV = 1.89%	R = 100.8%; CV = 2.78%	R = 98.8%; CV = 1.98%
for 150% standard added		R = 99.2%; CV = 2.46%	R = 101.6%; CV = 2.24%	R = 99.1%; CV = 1.78%
Quantity of Precision (CV, [%]) *n* = 3				
Interday	0.10 µg/spot	1.87	2.13	1.64
	0.50 µg/spot	2.13	0.89	1.89
	0.90 µg/spot	2.56	0.99	1.05
Intraday	0.10 µg/spot	1.45	1.78	1.56
	0.50 µg/spot	2.45	2.13	1.91
	0.90 µg/spot	2.81	1.89	1.08
Robustness		Robust	Robust	Robust

**Table 5:** Method validation data for the quantitative determination of diclofenac sodium by NP-TLC with densitometry after 2D separation using two mobile phases: first chloroform–methanol–ammonia (36:4:0.60 *v*/*v*/*v*) and second cyclohexane–chloroform–methanol–glacial acetic acid (6:3:0.5:0.5 *v*/*v*/*v*/*v*).

Method Characteristic	Results	
Specificity	Specific	
Range [μg/spot]	5.00–15.00	
Linearity	A = 6880.4 (±51.8) + 278.4 (±4.9) ∙ x *n* = 11; r = 0.998; s = 51.8; F = 3181.9; *p* < 0.0001	
Limit of detection (LOD) [μg/spot]	0.61	
Limit of quantification (LOQ) [μg/spot]	1.84	
For tablets Accuracy and precision		
Accuracy (*n* = 6)		
for 50% standard added (*n* = 6)	R = 103.1%; CV = 2.78%	
for 100% standard added (*n* = 6)	R = 98.5%; CV = 2.54%	
for 150% standard added (*n* = 6)	R = 98.4%; CV = 2.12%	
	Quantity of Precision (CV, [%]) *n* = 3	
	6.00 μg/spot	1.13
*Interday*	10.00 μg/spot	2.42
	14.00 μg/spot	1.88
	6.00 μg/spot	2.45
*Intraday*	10.00 μg/spot	2.78
	14.00 μg/spot	2.38
Robustness	Robust	

**Table 6:** Robustness of the proposed methods (*n* = 5).

Parameter	% RSD of Peak Area of			
	Diclofenac in DicloDuo Combi	Omeprazole in		
		DicloDuo Combi	Omeprazole Genoptim SPH	Bioprazol Bio Max
Chromatographic plates 1.05570 and 1.05554	1.11%	0.95%	1.13%	1.29%
Mobile phase volume (50 mL ± 5%)	0.89%	0.85%	0.88%	0.96%
Temperature of the activation of the plates at 120 (±5) °C	1.12%	1.13%	1.04%	1.38%
Development distance (±5 mm)	1.89%	0.97%	1.46%	1.29%
Time od saturation (±5 min)	1.28%	1.33%	1.12%	0.99%

**Table 7:** The statistical data concerning the results of the quantitative determination of omeprazole and diclofenac sodium in commercial simple and combined pharmaceutical capsules examined by elaborated NP-TLC with densitometry methods.

Number of Analysis	Pharmaceutical Preparation			
	DicloDuo Combi		Omeprazole Genoptim SPH	Bioprazol Bio Max
	Diclofenac Sodium	Omeprazole	Omeprazole	Omeprazole
1	73.9	19.1	20.8	18.6
2	74.2	18.8	21.1	20.5
3	72.9	19.6	20.5	20.7
4	74.5	19.2	19.7	20.1
5	73.4	19.0	20.7	20.9
6	74.9	18.9	21.2	20.4
Average amount [mg/capsule]	74.0	19.1	20.7	20.2
The label claim [mg/capsule]	75	20	20	20
Standard deviation (SD)	0.73	0.28	0.54	0.83
Coefficient of variation [CV%]	0.53	0.08	0.29	0.69
Confidence interval of arithmetic mean with confidence level equal 95%	μ = 74.0 ± 0.7	μ = 19.1 ± 0.3	μ = 20.7 ± 0.5	μ = 20.2 ± 0.8
Amount (%) in relations to the label claim	98.7	95.5	103.5	101.0

**Table 8:** Comparison of the limit of detection of omeprazole and diclofenac sodium determined in different pharmaceutical preparations.

Analytical Method	LOD	Ref.
Omeprazole		
TLC	12.7 ng/spot	[15]
TLC	0.961 ng/spot	[20]
TLC	0.020 µg/spot	[24]
TLC	0.009 µg/spot (9 ng/spot) 0.18 µg/mL	in this work
HPTLC	0.005 µg/spot	[18]
HPTLC	0.099 µg/spot	[27]
HPTLC	0.074 µg/spot	[17]
HPTLC	0.010 µg/spot	[19]
HPTLC	2.64 ng/spot	[21]
HPTLC	4.68 ng/spot	[22]
HPTLC	40.83 ng/spot	[30]
HP-TLC	7.9 ng/spot	[25]
HPLC-UV	0.4 µg/mL	[29]
RP-HPLC	131.27 ng/mL	[30]
RP-HPLC	0.076 µg/mL	[33]
RP-HPLC	0.0712 μg/mL	[34]
UHPLC	1.48 µg/mL	[24]
RP-HPLC	0.54 µg/mL	[39]
RP-HPLC	0.06 µg/mL	[31]
UV-Spectrophotometric	0.105 µg/mL	[41]
Spectrophotometric	0.033 µg/mL	[43]
Diclofenac sodium		
TLC	0.0107 µg/spot	[15]
TLC	0.28 µg/spot	[48]
TLC	1 µg/mL	[60]
TLC	0.61 µg/spot (122 µg/mL	in this work
RP-HPLC	0.239 μg/mL	[34]
RP-HPLC	0.011 µg/mL	[38]
GC–MS	0.15 μg/mL	[46]
linear sweep voltammetry (LSV)	4.8 μg/mL	[46]
UV-Spectrophotometric	0.048 µg/mL	[41]

## Data Availability

Data is contained within the article and Supplementary Material.

## Supplementary Materials

The following supporting information can be downloaded at https://www.mdpi.com/article/10.3390/ph15081016/s1. Figure S1. Densitogram of diclofenac sodium, the solution of which after UV irradiation was separated on silica gel using the mobile phase: chloroform–methanol–ammonia (36:4:0.60, *v*/*v*/*v*); Figure S2. densitogram of diclofenac sodium in alkaline solution, which after heating was separated on silica gel using the mobile phase: chloroform–methanol–ammonia (36:4:0.60, *v*/*v*/*v*); Figure S3. Densitogram of diclofenac sodium in a solution with the addition of hydrogen peroxide, which after heating was separated on silica gel using the mobile phase: chloroform–methanol–ammonia (36:4:0.6, *v*/*v*/*v*); Figure S4. Densitogram of diclofenac sodium in acidic solution, which after heating was separated on silica gel using the mobile phase: chloroform–methanol–ammonia (36:4:0.60, *v*/*v*/*v*); Figure S5. Densitogram of a standard solution of diclofenac sodium that was separated on silica gel using the mobile phase: chloroform–methanol–ammonia (36:4:0.60, *v*/*v*/*v*); Figure S6. Densitogram of omeprazole in alkaline solution, which after heating was separated on silica gel using the mobile phase: chloroform–methanol–ammonia (36:4:0.60, *v*/*v*/*v*), where O is omeprazole, A omeprazole-related compound A, and P unidentified omeprazole degradation products; Figure S7. Densitogram of omeprazole in solution with the addition of hydrogen peroxide, which after heating was separated on silica gel using the mobile phase: chloroform–methanol–ammonia 36:4:0.60, *v*/*v*/*v*); where O is omeprazole, A omeprazole-related compound A, and P unidentified omeprazole degradation products; Figure S8. Densitogram of omeprazole in a solution with the addition of physiological saline, which after heating was separated on silica gel using the mobile phase: chloroform–methanol–ammonia 36:4: 0.60, *v*/*v*/*v*); where: O is omeprazole, and P1 and P2 are unidentified omeprazole degradation products; Figure S9. Densitogram of omeprazole solution in methanol, which after UV irradiation was separated on silica gel using the mobile phase: chloroform–methanol–ammonia (36:4:0.60, *v*/*v*/*v*); where O is omeprazole and P1–P3 unidentified omeprazole degradation products; Figure S10. Densitogram of omeprazole derived from an extract of omeprazole genoptim SPH, which was analyzed on silica gel using the mobile phase: chloroform–methanol–ammonia (36:4:0.60, *v*/*v*/*v*); Figure S11. Densitogram of omeprazole standard, which was analyzed on silica gel using the mobile phase: chloroform–methanol–ammonia (36:4:0.60, *v*/*v*/*v*); Figure S12. Comparison of the spectrodensitograms of the omeprazole standard and omeprazole from the DicloDuo Combi combined preparation; Figure S13. Comparison of the spectrodensitograms of the diclofenac standard and diclofenac from the DicloDuo Combi combined preparation; Figure S14. Calibration plot (A) and plot of residuals (B) for omeprazole in the linear working range (mobile phase: chloroform–methanol–ammonia 36:4:0.60, *v*/*v*/*v*)*;* Figure S15. Calibration plot (A) and plot of residuals (B) for diclofenac sodium in the linear working range after 2D bidirectional development of the chromatography plate (first mobile phase: chloroform–methanol–ammonia (36:4:0.60, *v*/*v*/*v*), second mobile phase: cyclohexane–chloroform–methanol–glacial acetic acid 6:3:0.5:0.5 *v*/*v*/*v*/*v*); Table S1. The relative percentage error in the determination of the area of the chromatographic bands of omeprazole and diclofenac sodium.

## Sample Availability

Samples of the compounds are available from the authors.
