# Supplementary material for: TLC–Densitometry for Determination of Omeprazole in Simple and Combined Pharmaceutical Preparations
Source: Pharmaceuticals (Basel). 2022 Aug 18;15(8):1016. doi: 10.3390/ph15081016 (PMC9416117; doi:10.3390/ph15081016)
Supplement: Supplementary file 1 [file pharmaceuticals-15-01016-s001.zip › pharmaceuticals-1824977-supplementary(1) - 17 August.pdf]

TLC–Densitometry for Determination of Omeprazole in Simple and Combined Pharmaceutical Preparations

Wioletta Parys* **and Alina Pyka-Pająk***

Department of Analytical Chemistry, Faculty of Pharmaceutical Sciences in Sosnowiec, Medical University of Silesia in Katowice, Jagiellońska 4, 41-200 Sosnowiec, Poland

* Correspondence: wparys@sum.edu.pl (W.P.); apyka@sum.edu.pl (A.P.-P.); Tel.: +48-32-364-15-34 (W.P.); +48-32-364-15-30 (A.P.-P.)

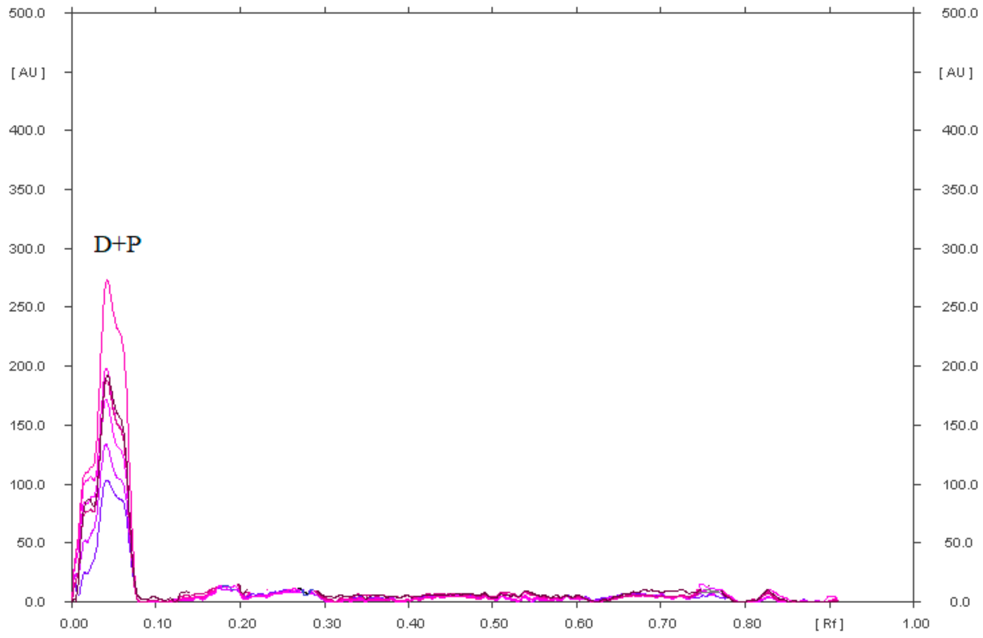

Figure S1. Densitogram of diclofenac sodium, the solution of which after UV irradiation was separated on silica gel using the mobile phase: chloroform+methanol+ammonia (36:4:0.60, v/v/v).

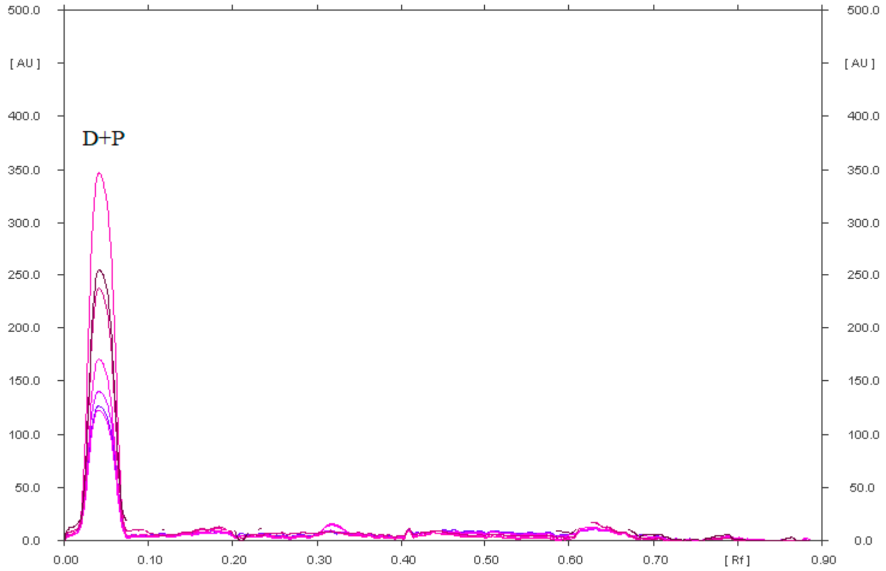

Figure S2. Densitogram of diclofenac sodium in alkaline solution, which after heating was separated on silica gel using the mobile phase: chloroform+methanol+ammonia (36:4:0.60, v/v/v).

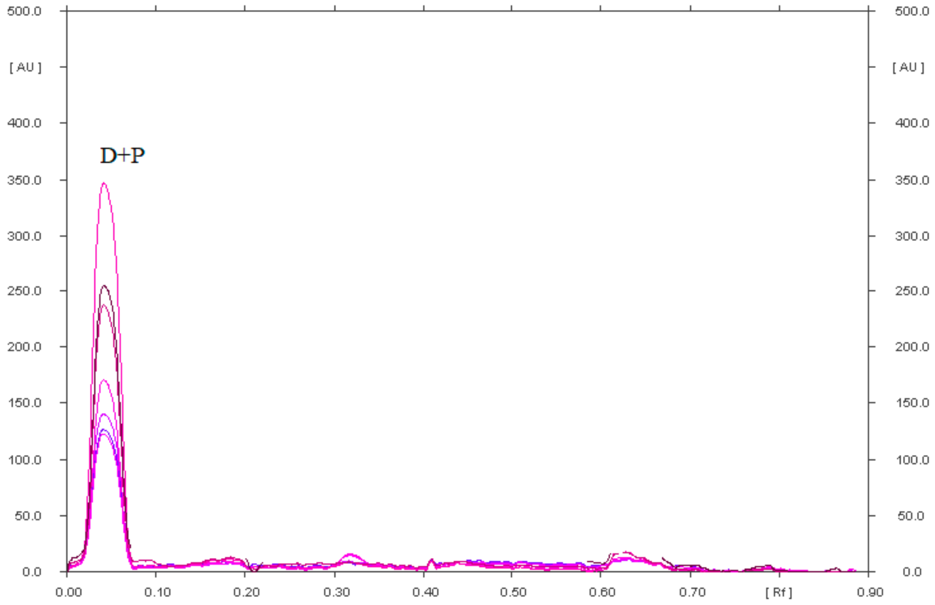

Figure S3. Densitogram of diclofenac sodium in a solution with the addition of hydrogen peroxide, which after heating was separated on silica gel using the mobile phase: chloroform+methanol+ammonia (36:4:0.6, v/v/v).

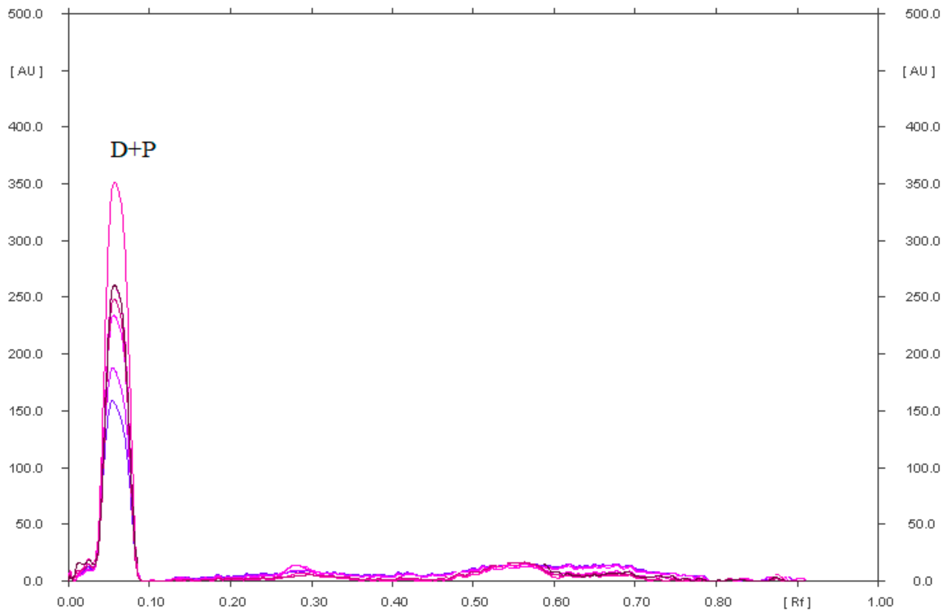

Figure S4. Densitogram of diclofenac sodium in acidic solution, which after heating was separated on silica gel using the mobile phase: chloroform+methanol+ammonia (36:4:0.60, v/v/v).

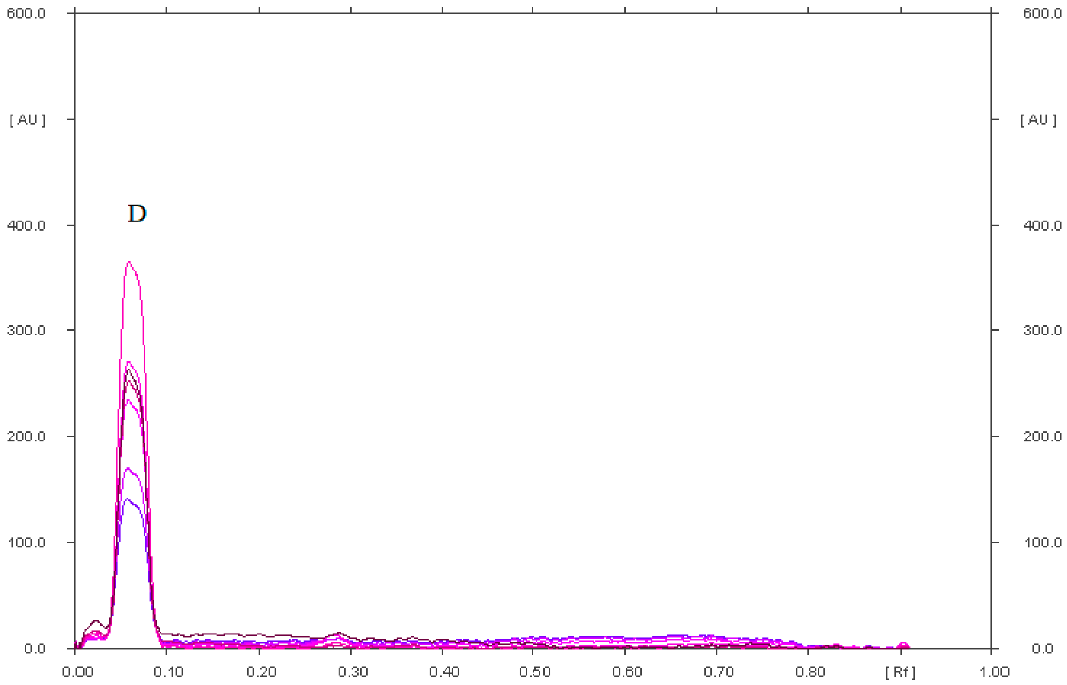

Figure S5. Densitogram of a standard solution of diclofenac sodium that was separated on silica gel using the mobile phase: chloroform+methanol+ammonia (36:4:0.60, v/v/v).

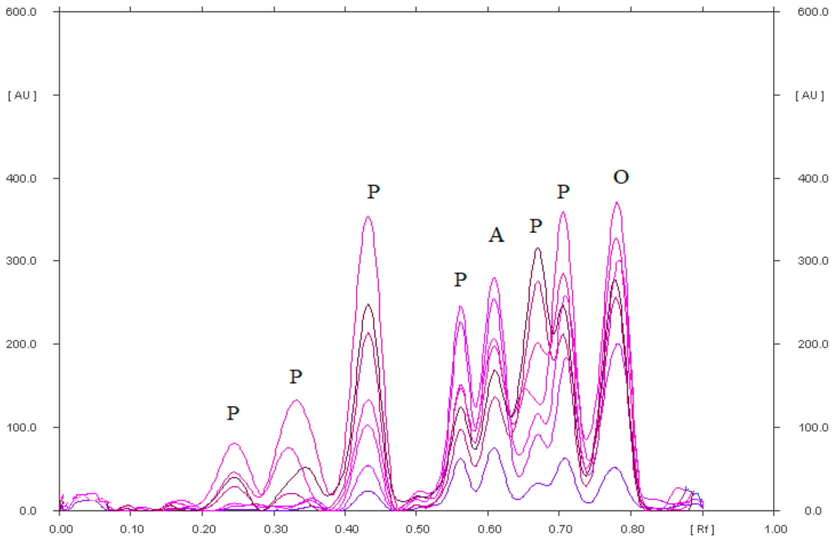

Figure S6. Densitogram of omeprazole in alkaline solution, which after heating was separated on silica gel using the mobile phase: chloroform+methanol+ammonia (36:4:0.60, v/v/v); where: O-omeprazole, A - omeprazole related compound A, P - unidentified omeprazole degradation products.

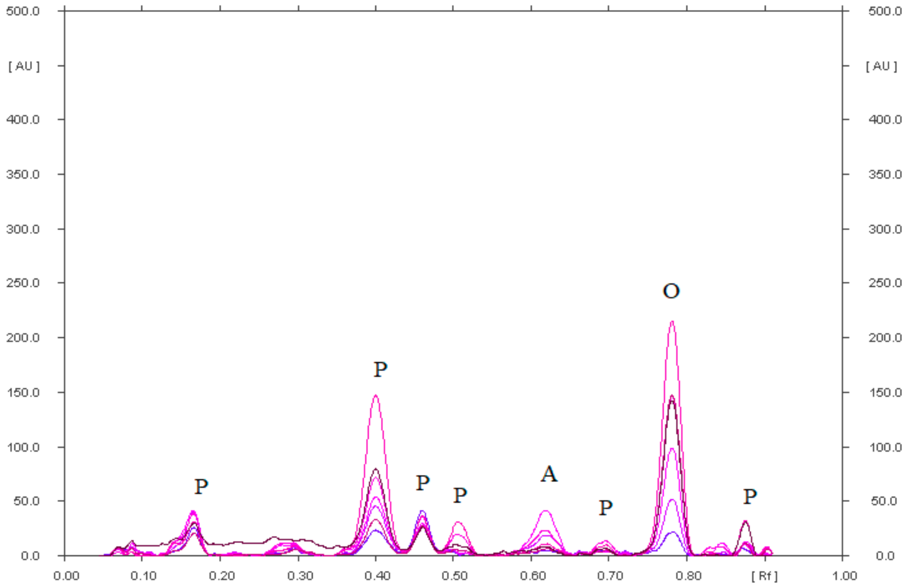

Figure S7. Densitogram of omeprazole in solution with the addition of hydrogen peroxide, which after heating was separated on silica gel using the mobile phase: chloroform+methanol+ammonia 36:4: 0.60, v/v/v); where: O-omeprazole, A - omeprazole related compound A, P - unidentified omeprazole degradation products.

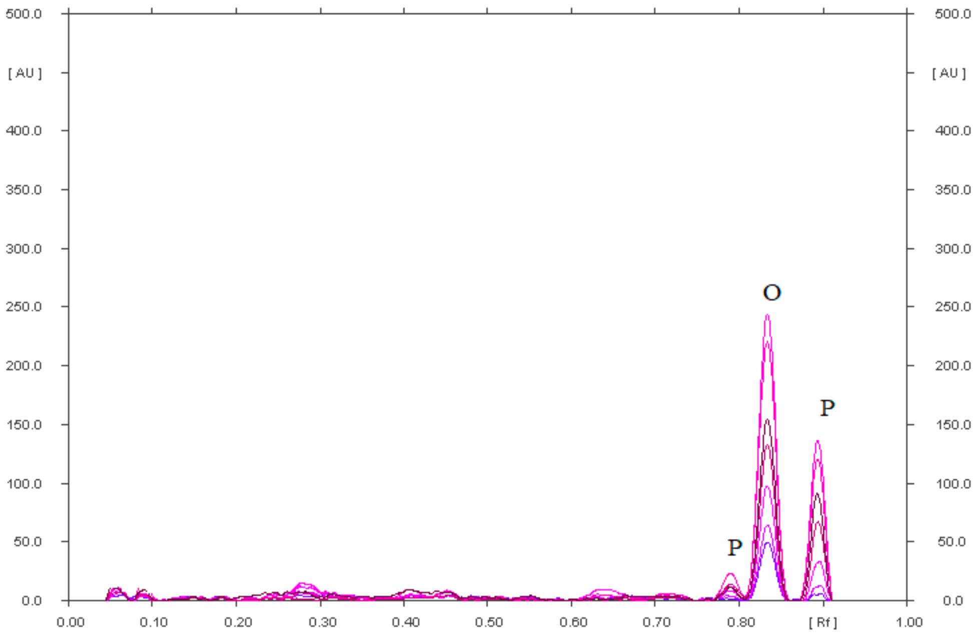

Figure S8. Densitogram of omeprazole in a solution with the addition of physiological saline, which after heating was separated on silica gel using the mobile phase: chloroform+methanol+ammonia 36:4: 0.60, v/v/v); where: O-omeprazole, and P1, P2 – unidentified omeprazole degradation products.

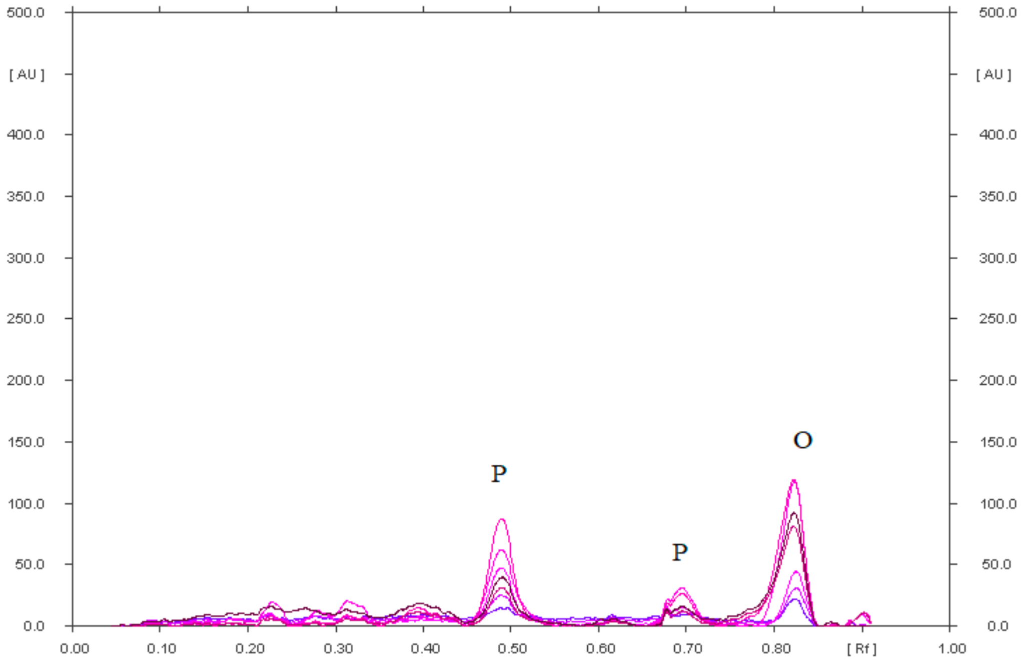

Figure S9. Densitogram of omeprazole solution in methanol, which after UV irradiation was separated on silica gel using the mobile phase: chloroform+methanol+ammonia (36:4:0.60, v/v/v); where: O-omeprazole and P1, P2, P3 - unidentified omeprazole degradation products.

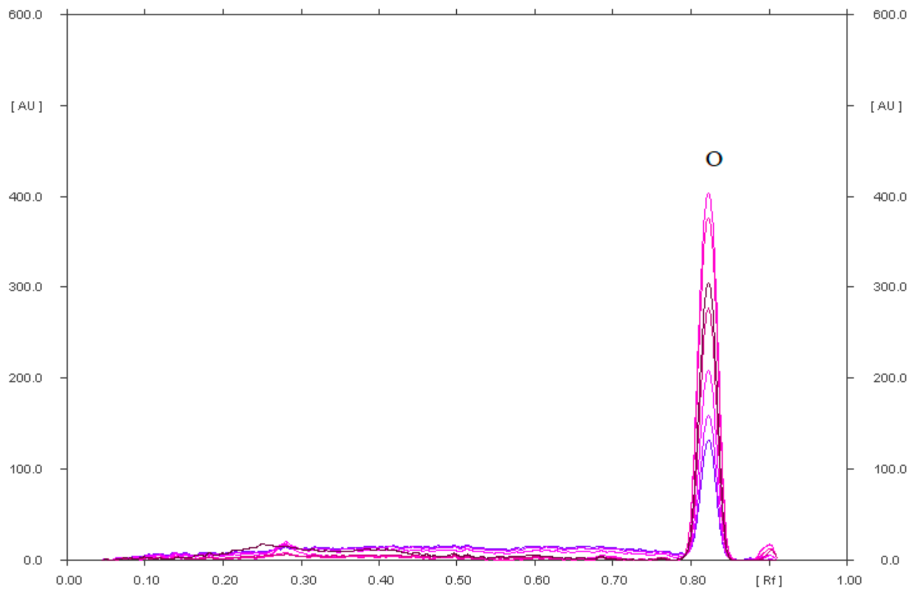

Figure S10. Densitogram of omeprazole derived from an extract of Omeprazole Genoptim SPH, which was analyzed on silica gel using the mobile phase: chloroform+methanol ammonia (36:4:0.60, v/v/v).

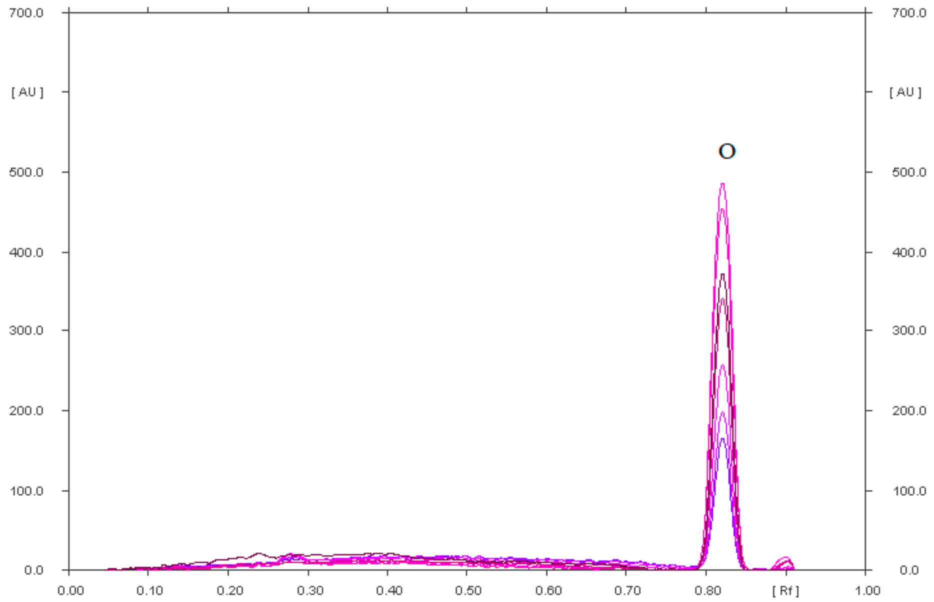

Figure S11. Densitogram of omeprazole standard, which was analyzed on silica gel using the mobile phase: chloroform+methanol+ammonia (36:4:0.60, v/v/v).

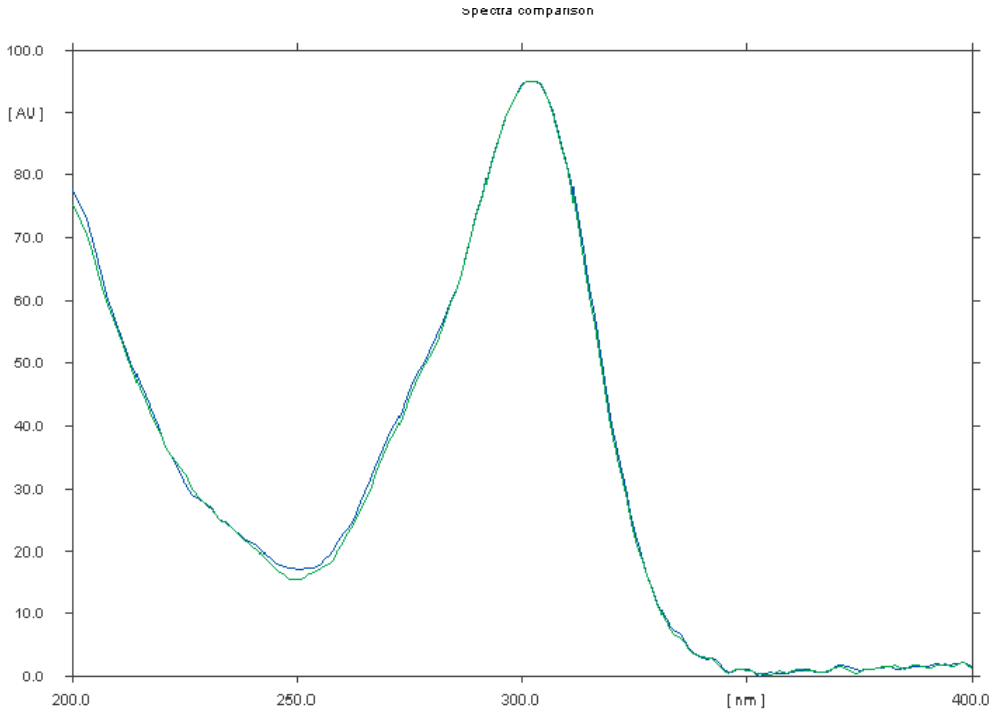

Figure S12. Comparison of the spectrodensitograms of the omeprazole standard and omeprazole from the DicloDuo Combi combined preparation.

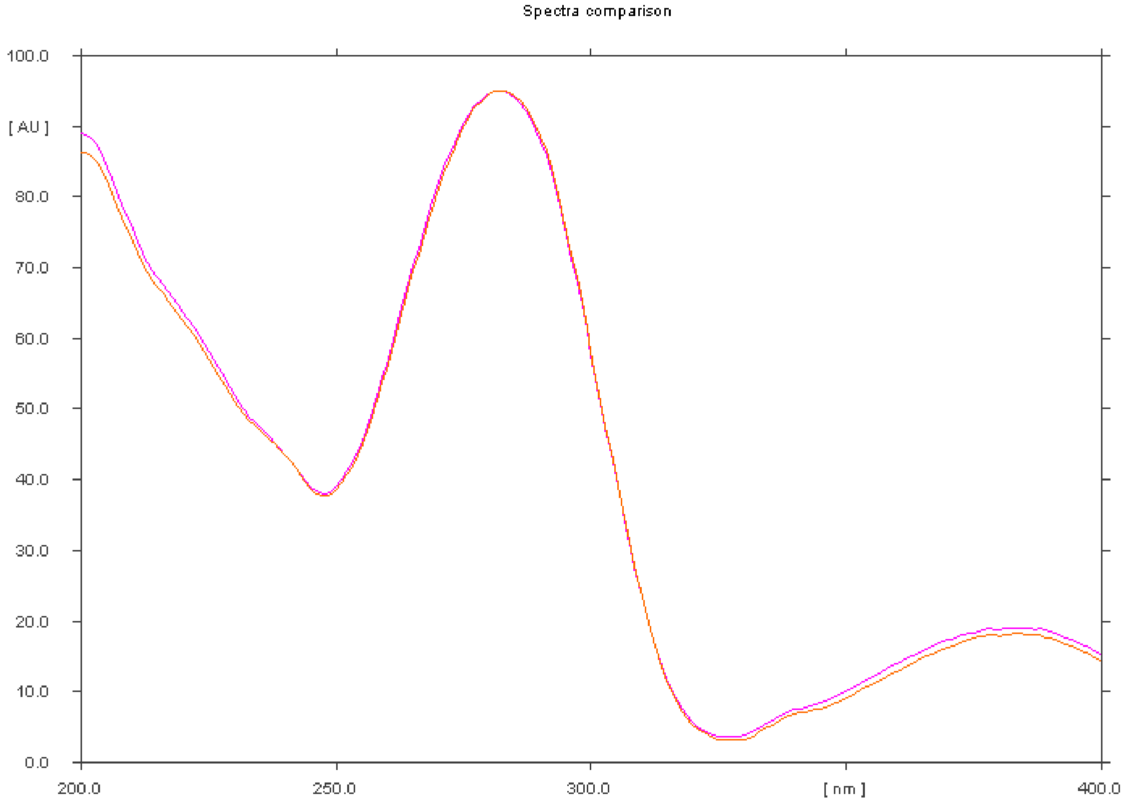

Figure S13. Comparison of the spectrodensitograms of the diclofenac standard and diclofenac from the DicloDuo Combi combined preparation.

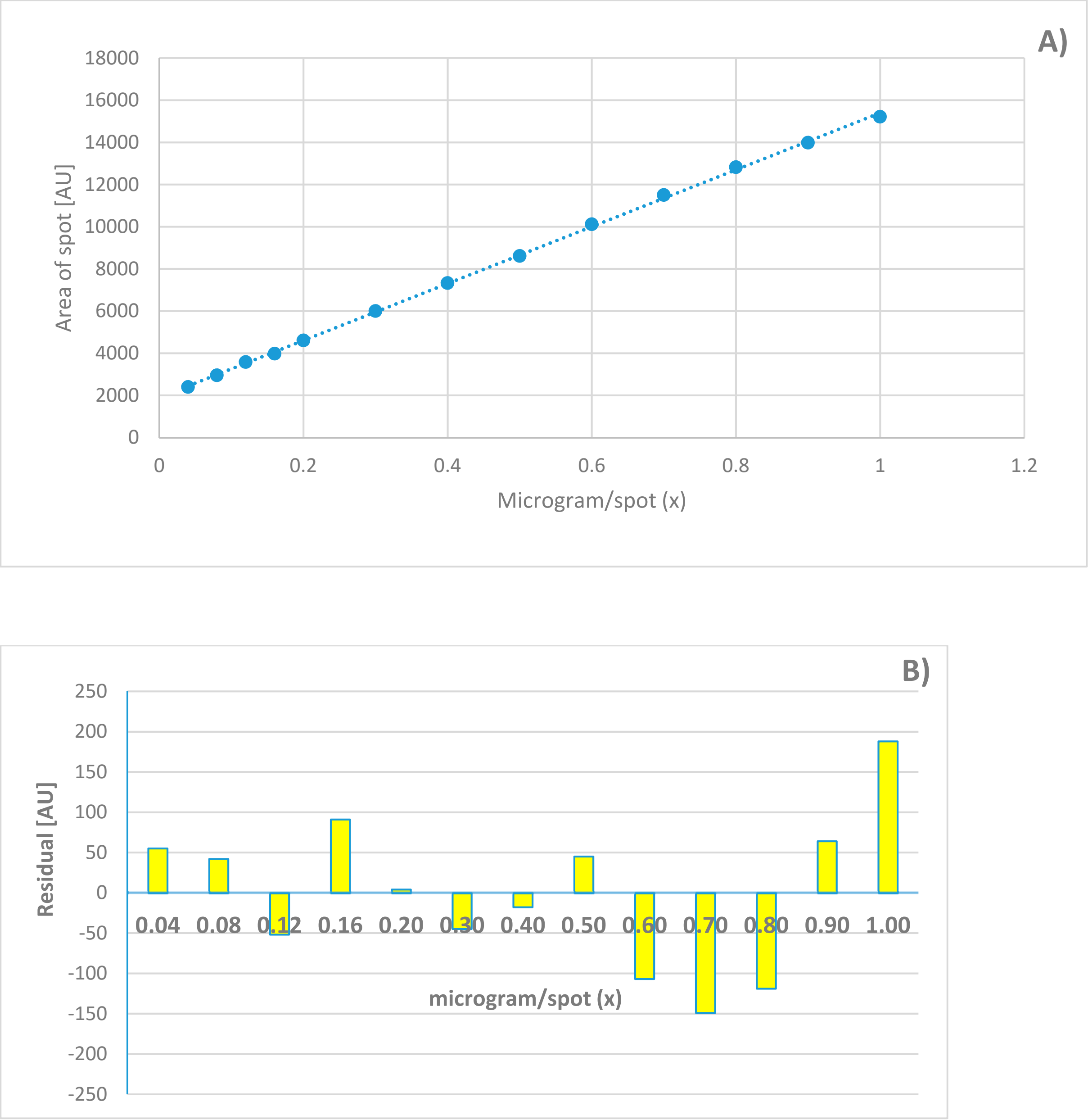

Figure S14. Calibration plot (A) and plot of residuals (B) for omeprazole in the linear working range (mobile phase: chloroform+methanol+ammonia 36:4:0.60, v/v/v).

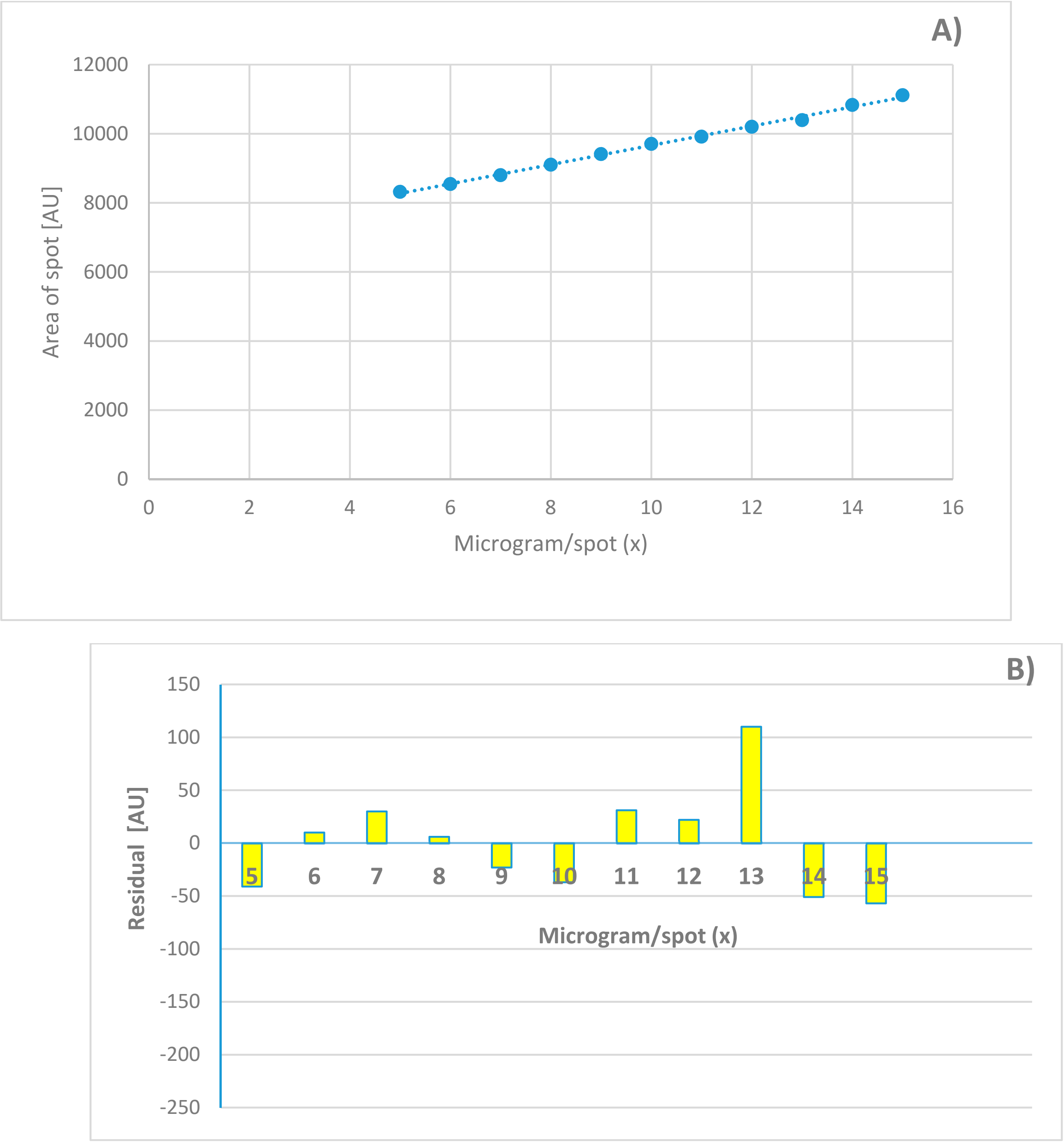

Figure S15. Calibration plot (A) and plot of residuals (B) for diclofenac sodium in the linear working range after 2D bidirectional development of the chromatography plate (first mobile phase: chloroform +methanol+ammonia (36:4:0.60, v/v/v); second mobile phase: cyclohexane+chloroform+methanol+ glacial acetic acid 6:3:0.5:0.5 v/v/v/v).

Table S1. The relative percentage error in the determination of the area of the chromatographic band of omeprazole and diclofenac sodium, respectively.

Omeprazole		Diclofenac sodium	
Analyte concentration [μg/spot]	The relative percentage error in the determination of the area of the chromatographic band	Analyte concentration [μg/spot]	the relative percentage error in the determination of the area of the chromatographic band
1	-1.24%	15	0.51%
0.9	-0.46%	14	0.48%
0.8	0.93%	13	-1.06%
0.7	1.30%	12	-0.21%
0.6	1.06%	11	-0.32%
0.5	-0.52%	10	0.38%
0.4	0.25%	9	0.25%
0.3	0.76%	8	-0.07%
0.2	-0.08%	7	-0.34%
0.16	-2.29%	6	-0.11%
0.12	1.45%	5	0.49%
0.08	-1.41%		
0.04	-2.30%		
